# Brockarchaeota, a novel archaeal phylum with unique and versatile carbon cycling pathways

**DOI:** 10.1038/s41467-021-22736-6

**Published:** 2021-04-23

**Authors:** Valerie De Anda, Lin-Xing Chen, Nina Dombrowski, Zheng-Shuang Hua, Hong-Chen Jiang, Jillian F. Banfield, Wen-Jun Li, Brett J. Baker

**Affiliations:** 1 Department of Marine Science, University of Texas Austin, Port Aransas, TX 78373 USA; 2 Department of Earth and Planetary Sciences, University of California, Berkeley, CA USA; 3 NIOZ, Royal Netherlands Institute for Sea Research, Department of Marine Microbiology and Biogeochemistry, and Utrecht University, Den Burg, Netherlands; 4 Department of Environmental Science and Engineering, University of Science and Technology of China, Hefei, PR China; 5 State Key Laboratory of Biogeology and Environmental Geology, China University of Geosciences, Wuhan, People’s Republic of China; 6 Department of Environmental Science, Policy and Management, University of California, Berkeley, CA USA; 7 State Key Laboratory of Biocontrol, Guangdong Provincial Key Laboratory of Plant Resources and Southern Marine Science and Engineering Guangdong Laboratory (Zhuhai), School of Life Sciences, Sun Yat-Sen University, Guangzhou, People’s Republic of China; 8 State Key Laboratory of Desert and Oasis Ecology, Xinjiang Institute of Ecology and Geography, Chinese Academy of Sciences, Urumqi, People’s Republic of China

**Keywords:** Microbial ecology, Taxonomy, Archaeal genomics, Metagenomics

## Abstract

Geothermal environments, such as hot springs and hydrothermal vents, are hotspots for carbon cycling and contain many poorly described microbial taxa. Here, we reconstructed 15 archaeal metagenome-assembled genomes (MAGs) from terrestrial hot spring sediments in China and deep-sea hydrothermal vent sediments in Guaymas Basin, Gulf of California. Phylogenetic analyses of these MAGs indicate that they form a distinct group within the TACK superphylum, and thus we propose their classification as a new phylum, ‘Brockarchaeota’, named after Thomas Brock for his seminal research in hot springs. Based on the MAG sequence information, we infer that some Brockarchaeota are uniquely capable of mediating non-methanogenic anaerobic methylotrophy, via the tetrahydrofolate methyl branch of the Wood-Ljungdahl pathway and reductive glycine pathway. The hydrothermal vent genotypes appear to be obligate fermenters of plant-derived polysaccharides that rely mostly on substrate-level phosphorylation, as they seem to lack most respiratory complexes. In contrast, hot spring lineages have alternate pathways to increase their ATP yield, including anaerobic methylotrophy of methanol and trimethylamine, and potentially use geothermally derived mercury, arsenic, or hydrogen. Their broad distribution and their apparent anaerobic metabolic versatility indicate that Brockarchaeota may occupy previously overlooked roles in anaerobic carbon cycling.

## Abstract (web summary)

Geothermal environments are hotspots for carbon cycling. Here, De Anda et al. reconstruct archaeal genomes from terrestrial and deep-sea geothermal sediments, and propose the classification of these microbes as a new phylum, ‘Brockarchaeota’, with unique metabolic capabilities including non-methanogenic anaerobic methylotrophy.

## Introduction

Advances in DNA sequencing and computational approaches have accelerated the reconstruction of metagenome assembled genomes (MAGs) from natural communities^1^. This approach has revealed many novel lineages on the tree of life and is advancing our understanding the ecological roles of uncultured microbes^1–3^. For example, many new archaeal phyla have been described from hot springs including Geoarchaeota^4^, Marsarchaeota^5^, Aigarchaeota^6^, and several Asgard phyla from deep-sea hydrothermal vents^7–12^. However, diversity surveys have demonstrated there are many novel taxa left to be explored^13^. Moreover, there are several gaps between our knowledge of active biogeochemical processes and the metabolic mechanisms and taxa mediating them. For example, the description of microbes mediating anaerobic methylotrophy is still limited, and it is unclear which non-methanogenic heterotrophs utilize methylated compounds on the anoxic seafloor^14^. Little is known about the microorganisms or pathways mediating this process^15^.

Methylotrophs are organisms that are capable of using simple organics including single-carbon (C1 e.g., methanol) and methylated (e.g., trimethylamine) compounds as a source of energy and carbon^16,17^. In nature, the most prevalent are compounds such as methanol and methylamines, which are derived from a variety of sources such as phytoplankton, plants, and the decay of organic matter^15,18,19^. As a result, they are ubiquitous in oceans and atmosphere and are important components of the global carbon and nitrogen cycles^15^. In oxic environments, methanol is converted to formaldehyde by the classical pyrroloquinoline quinone (PQQ)-linked methanol dehydrogenase pathway found in aerobic methylotrophs^15,18^. In anoxic settings, these compounds are used as substrate for methylotrophic-methanogenesis^20–23^ and sulfate reduction^24^. Anaerobic methylotrophs utilize the methyltransferase system (MT) to break and transfer the methyl residue to coenzyme M (in the case of methanogens) or tetrahydrofolate (H_4_F) (in acetogens and sulfate reducers)^20–24^ and conserved energy via the Wood–Ljungdahl pathway (WLP). Methylotrophic archaea include methanogenic orders (in Euryarchaeota): *Methanosarcinales*, *Methanobacteriales*, *Methanomassiliicoccales*, and the recently discovered uncultured methylotrophic phylum, *Verstraetearchaeota*^20^. Methylotrophy has not been described in archaeal lineages outside of these methanogenic groups.

Here we describe a new archaeal phylum, the Brockarchaeota, whose members are metabolically versatile and can be found in geothermal environments around the world. The Brockarchaeota appear to possess diverse pathways for carbon cycling including fermentation of complex organic carbon compounds, anaerobic methylotrophy, and chemolithotrophy.

## Results

### Genomic reconstruction

Metagenomic sequencing, assembly, and binning of sediments from seven terrestrial hot springs in Tibet (up to 70°C) and Tengchong Yunnan, China (up to 86 °C) and deep sea hydrothermally heated Guaymas Basin (GB) sediments (10–34°C) resulted in the reconstruction of 15 draft metagenome-assembled genomes (MAGs) estimated to be 67–92% complete (Table 1). These MAGs range from 0.78 to 2.32 Mbp (average 1.47 Mbp) (Table 1). The two MAGs from GB (B48_G17 and B27_G9, temperature 33.62 and 10.4°C, respectively) were originally designated as “GB-AP1” in a prior study^25^. Although the GB genomes were obtained from lower temperatures these sediments experience increases in temperature due to hydrothermal circulation^25^. Thus, the organisms from which these genomes were derived likely prefer hot geothermal ecosystems, and anoxic conditions (Supplementary Data 1). All of these MAGs are rare members of the communities they were recovered from, presenting 0.1 to 9% of the genomic reads (Supplementary Data 2).

**Table 1:** General information on the fifteen novel Brockarchaeota MAGs.

Genome	Origin	Size (Mb)	Scaffolds (number)	Protein coding genes (number)	Predicted size(Mb)	GC (%)	Compl.(%)	Red.(%)	Largest scaffold(bp)	Strain hetero
B48_G17	Deep sea sediment (12–15 cm, 33.6 °C) Vent 2 in Dombrowski et al. ^25^	0.78	110	911	0.92	51.06	83.1	0.93	49,468	100
B27_G9	Deep sea sediment (0–3 cm, 10.4 °C) Vent 2 in Dombrowski et al. ^25^	0.99	223	1341	1.31	42.30	67	1.46	17,768	0
DRTY7-35_44	Hot spring sediment DiReTiYanQu-7 (collected in Jan, 2016) in Tengchong county, Yunnan, China (55.8 °C)	0.83	130	910	0.95	34.17	84	1.94	23,553	0
QC4_43	Hot spring QuCai village, Tibet, China (69.5 °C)	1.18	36	1332	1.42	43.17	80	2.43	135,049	0
QC4_48	Hot spring QuCai village, Tibet, China (69.5 °C)	1.66	86	1793	1.92	47.78	84.47	1.29	78,542	0
GD2_1	Hot spring GuDui geothermal area, Tibet, China (61.8 °C)	2.14	156	2326	2.37	47.13	89	1.94	82,330	0
QZM_A2	Hot spring QuZhuoMu village, Tibet, China (63.1 °C)	2.19	173	2447	2.59	47.44	82	1.94	96,973	0
QZM_A3	Hot spring QuZhuoMu village, Tibet, China (62.9 °C)	1.96	216	2270	2.38	47.89	78.64	3.88	70,810	0
DRTY-1.18	Hot spring sediment DiReTiYanQu-1 (collected in May, 2017) in Tengchong county, Yunnan, China (67 °C)	1.62	55	1582	1.81	34.71	88	1.94	117,455	0
DRTY-6.200	Hot spring sediment DiReTiYanQu-6 (collected in May, 2017) in Tengchong county, Yunnan, China (60 °C)	0.55	129	663	0.78	34.24	57	0	11,975	0
DRTY-6.80	Hot spring sediment DiReTiYanQu-6 (collected in May, 2017) in Tengchong county, Yunnan, China (60 °C)	2.32	55	2264	2.61	47.51	87	0	266,602	0
DRTY-7.37	Hot spring sediment DiReTiYanQu-7 (collected in Jan, 2016) in Tengchong county, Yunnan, China (55.8 °C)	1.03	189	1131	1.17	34.76	87	0.97	23,662	0
JZ-1.89	Hot spring sediment Jinze-1 (collected in May, 2017) in Tengchong county, Yunnan, China (86.5 °C)	1.48	88	1680	2.20	42.38	51	0.97	147,883	0
JZ-2.136	Hot spring sediment Jinze-2 (collected in May, 2017) in Tengchong county, Yunnan, China (63 °C)	2.04	214	2235	2.36	57.55	84	0.97	40,656	0
JZ-2.4	Hot spring sediment Jinze-2 (collected in Jan, 2016) in Tengchong county, Yunnan, China (75 °C)	1.30	61	1412	1.40	41.33	92	0	130,091	0

Genome ID, origin, number of scaffolds, number of protein-coding genes, guanine-cytosine (GC) content, estimated completeness (Compl.), estimated gene redundancy (Red.), and strain heterogeneity (Strain hetero.) are shown (Extended genomic statistics are found in Supplementary Data 1).

### Phylogeny and distribution in nature

**Fig. 1:**
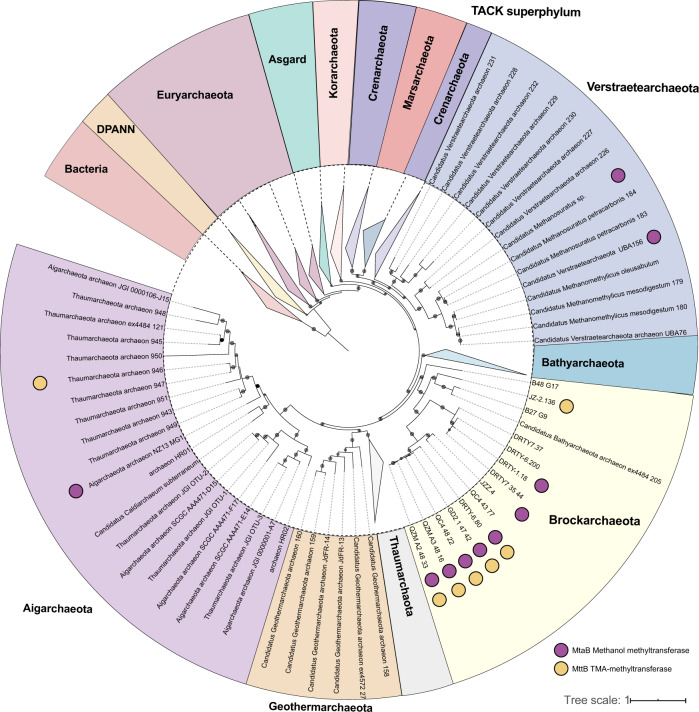
Comparison of phylogeny and distribution of methyltransferase system in Brockarchaeota and other members of TACK superphylum. Phylogeny generated using IQtree v1.6.11 using a concatenation of 37 conserved single-copy proteins using phylosift^63^ with a best fit LG + F + R10 model selected using the Bayesian Information Criterion (BIC), bootstrap values were calculated using non-parametric bootstrapping with 1,000 replicates (represented by gray circles, only bootstrap >70 are shown). Raw tree file is available in Supplementary Data 5; interactive version of the tree can be found at https://itol.embl.de/tree/7618619231376741590439752#. The presence methanol methyltransferase MtaB (PF12176) and trimethylamine methyltransferase MttB (PF06253) are shown in the outer circles. The annotation was conducted with MEBS^72^ details can be found in Supplementary Data 6.

**Fig. 2:**
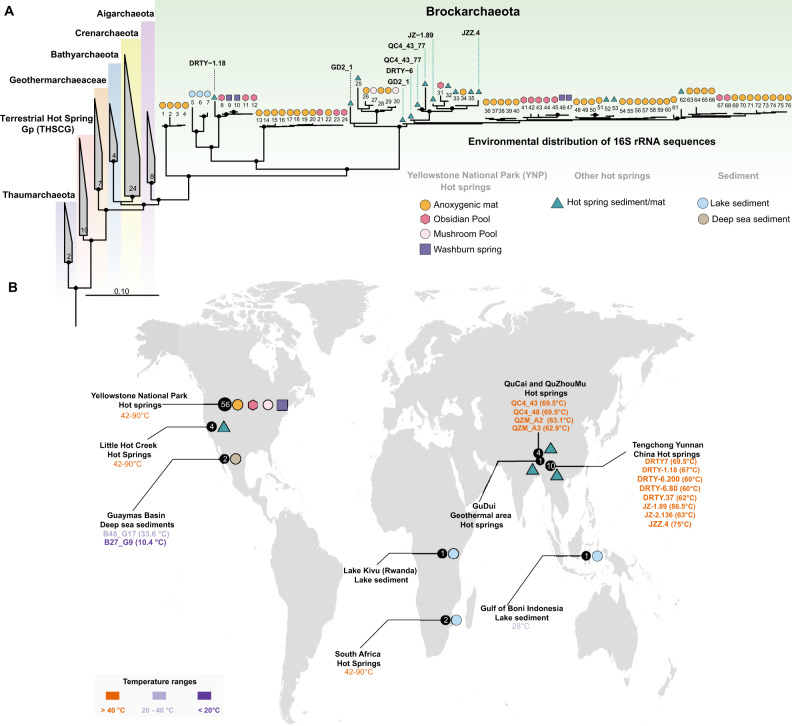
Location of samples from which Brockarchaeota genomes and 16S rRNA gene sequences have been recovered. **A** 16S rRNA gene tree of sequences derived from metagenomic and rRNA-based diversity surveys (NCBI accessions EU924237, KX213943, and KX213897). The eight complete 16S rRNA gene sequences of Brockarchaeota genomes described in this study are shown with their respective names and can be found in Supplementary data 7. Black circles in the tree represent bootstrap values using RAxML v8.2.10 with 100 replicates in the ARB software package v. 2.5b. Environmental information of each sequence were obtained from Integrated Microbial Genomes and Microbiomes database. Detailed information about the numbers displayed in the tree branches with their corresponding environmental information is described in Supplementary Data 8. **B** Geographic localization from which Brockarchaeota sequences were obtained. The size of the circle corresponds to the total number of Brockarchaeota-related sequences in each geographic location. The specific MAGs obtained in this study are shown in the map according to their temperature range. World map shape with the geographical coordinates of the Brockarchaeota 16S rRNA sequences shown in panel **A**, was created using ggmap^80^.

Phylogenetic analyses of these MAGs based on a concatenated alignment of 37 conserved proteins (see Methods), revealed they form a distinct group within the TACK superphylum, basal to *Aigarchaeota* and *Thaumarchaeota* (Fig. 1 and Supplementary Data 3). A comparison of average amino acid identities (AAI) across 250 available TACK genomes (Supplementary Data 4), revealed that Brockarchaeota are distinct from neighboring phyla (*Geoarchaeota*, *Aigarchaeota*, and *Thaumarchaeota*) and share up to 99% genome-wide nucleotide similarity to one another (Supplementary Fig. 1 and Supplementary Data 5). The two GB MAGs (B48_G17 and B27_G9) are distinct from the hot springs at the AAI level (<50% similar to each other), and <45% AAI to members of *Geoarchaeota*, *Thaumarchaeota*, and *Aigarchaeota*, which is consistent with their phylogenetic placement. Phylogeny of 16S rRNA genes also indicated that Brockarchaeota do not fall within any described archaeal phyla (Fig. 2A), with <78% similarity to other TACK members. Together, these results support the classification of these MAGs as a new phylum. We propose that the phylum be named “Brockarchaeota”, after Thomas Brock, an American microbiologist known for his groundbreaking research in hot springs microbiology.

Interestingly, only three 16S rRNA gene sequences with similarity (92–96%) to Brockarchaeota sequences have been described in PCR-based surveys, highlighting the inherent bias for primer choice in diversity studies. Therefore, we searched publicly available metagenomic databases to examine the geographic distribution of this phylum. Notably, we almost exclusively found 16S rRNA gene sequences related to Brockarchaeota in sequence data generated from other hot springs from around the world (China, USA, South Africa; Fig. 2A), revealing Brockarchaeota are globally distributed in hot springs (Fig. 2B). Three sequences, which cluster together, were recovered from lake sediments in Rwanda and the Gulf of Boni in Indonesia (28°C) (see Supplementary Data 8), suggesting that some Brockarchaeota are mesophilic as well.

### Unique anaerobic methylotrophic pathways

**Fig. 3:**
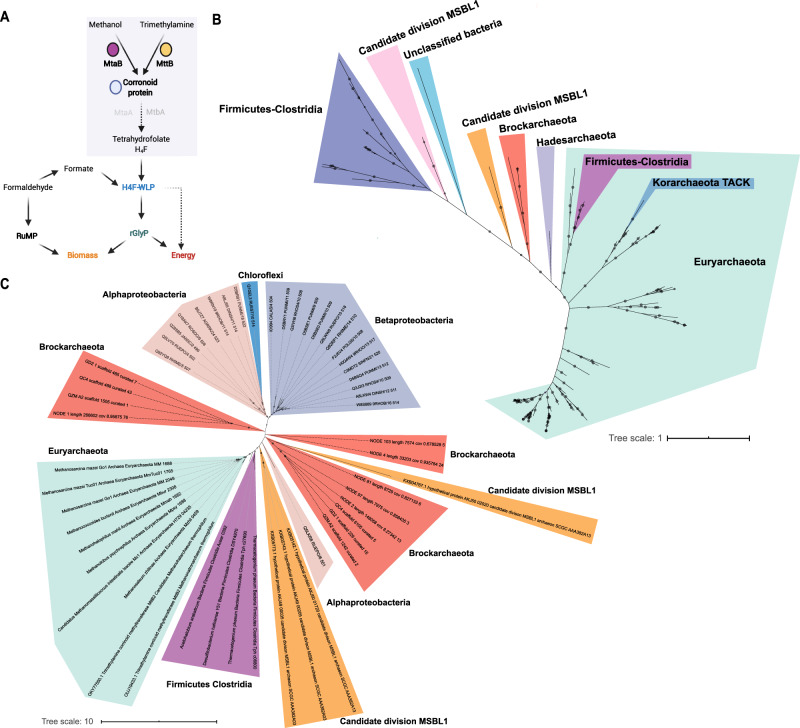
Proposed mechanism and phylogeny of the main enzymes of the methyltransferase system MTS. **A** Schematic model of the hypothesized methylotrophic metabolism in Brockarchaeota. C1 units of methanol and trimethylamine TMA are transferred to tetrahydrofolate (H_4_F) via Methyltransferase System (MTS) to be ultimately converted to biomass or energy via convergent assimilatory routes for formaldehyde and formate reductive glycine pathway (rGlyP) and ribulose monophosphate pathway (RuMP). See more details in the text. **B** Phylogeny of methanol methyltransferases B subunit (MtaB). A total of seven methanol methyltransferase proteins were detected in Brockarchaeota and were aligned with 101 PFAM (PF12176) sequences, and 104 references obtained from Muñoz-Velasco et al.^28^ and one Korarchaeota sequence from McKay et al.^27^. **C** Phylogeny of trimethylamine methyltransferases A subunit (MttB). A total of 12 methanol methyltransferase proteins were detected in Brockarchaeota MAGs and were aligned with 24 PFAM (PF6253) sequences, and 18 references from Muñoz-Velasco et al.^28^. The alignments were done using MAFFT v7.450 (default parameters) and refined with MUSCLE v3.8.425 (default parameters), then masked (50% gaps) in Geneious Prime 2020.0.5. The trees were generated using a maximum likelihood-based approach using RAxML v8.2.10 (Gamma+LG+F model, raxmlHPC-PTHREADS-AVX -f a -m PROTGAMMAAUTO -N autoMRE -p 12345 -x 12345 -s).

To begin to understand the metabolism of the Brockarchaeota we compared the predicted proteins encoded by these genomes with a variety of functional databases (see Methods). This revealed a potential unique type of anaerobic methylotrophic metabolism not yet described in archaea. They contain the methyltransferase system (MT), that has been shown to be essential for anaerobic methylotrophy^26^ and is composed of two key steps. First, specific methyltransferases break C–R bonds in a variety of substrates (MtaB for methanol, MtmB for monomethylamine, MtbB for dimethylamine, and MttB for trimethylamine) and transfer the methyl moieties to subunit MtaC. The second methyltransferase (MtaA for methanol, MtbA for methylamines), transfers the methyl-group from the corrinoid protein to coenzyme M in methanogens, or tetrahydrofolate in acetogens^24^. Brockarchaeota from hot springs encode proteins predicted to be methanol-cobalamin methyltransferases (MtaB) and trimethylamine-corrinoid protein methyltransferase (MttB) for the utilization of methanol and trimethylamine (TMA), respectively. B12-binding corrinoid protein genes are always colocalized with *mtaB* (Supplementary Data 9), suggesting a co-transcription of both subunits of the methyltransferase complex. Due to the lack of MtaA proteins, we suggest that another undescribed protein may be involved in the transfer of the methylated compound from the corrinoid protein to tetrahydrofolate (see details in Supplementary Discussion). Searches for MtaB and MttB proteins within TACK superphylum indicate methanol-MT system is a unique feature of Brockarchaeota (Fig. 1). Phylogenetic analyses of MtaB and MttB revealed Brockarchaeota form new branches within methanol and TMA methyltransferases (Fig. 3).

These results expand the distribution of anaerobic methylotrophy. Anaerobic methanol-utilization has only been described in Euryarchaeota and TACK (Verstraetaearchaeota^20^, Korarchaeota^27^) archaea, and some bacteria (Firmicutes and Deltaproteobacteria)^28^. However, Brockarchaeota genomes do not possess the key genes for methanogenesis including methyl-coenzyme M reductase (MCR) found in other archaea that have the MT system (Supplementary Data 10). To ensure that Brockarchaeota *mcr* genes were not overlooked, we searched metagenomic datasets from each of their communities and did not find any (See Supplementary Data 11). In addition to lacking Mcr, they lack key enzymes of the H_4_MPT methyl branch of the WLP, involved in the transfer and reduction of C1 moieties for methane production. The H_4_MPT is the natural pathway for degrading methylated compounds in methanogenic archaea^20^ and a key module for methylotrophy, carbon assimilation, formaldehyde detoxification in methylotrophic bacteria^16,17,29,30^. Furthermore, they lack the key enzyme for the carbonyl branch for the WLP, the CO-dehydrogenase−acetyl-CoA-synthase complex CODH/ACS^31^. Finally, Brockarchaeota do not encode the pyrroloquinoline quinone (PQQ)-linked methanol dehydrogenase pathway for aerobic methylotrophy (Supplementary Fig. 2) and recently found in deep-sea anaerobic bacteria^24^.

The lack of previously described methylotrophic pathways raises the question about how C1 compounds are assimilated in Brockarchaeota. Our findings suggest they metabolize formaldehyde, methanol, and trimethylamine via convergent assimilatory pathways for biosynthesis and energy conservation. A detailed comparative analysis suggests that Brockarchaeota assimilate methanol and TMA via the convergent action of the tetrahydrofolate (H_4_F) methyl branch of the WL pathway (H_4_F-WLP) and reductive glycine pathway (rGlyP).

### Formaldehyde assimilation

Brockarchaeota MAGs (B48_G17, DRTY7_35_44, DRT-1.18, JZ-2.136, DRTY7.37) encode both key enzymes of the ribulose monophosphate pathway (RuMP): 3-hexulose-6-phosphate synthase (HPS) and 6-phospho-3-hexuloisomerase (PHI). The RuMP pathway was originally found in methylotrophic bacteria that are able to use C1 compounds as a sole source of carbon and energy; however, it is currently recognized as a widespread prokaryotic pathway for formaldehyde fixation and detoxification^32^. The RuMP pathway functions as a highly efficient system for trapping free formaldehyde at relatively low concentrations. The presence of HPS and PHI in Brockarchaeota genomes suggests that formaldehyde can be fixed and detoxified via the RuMP pathway. Further oxidation of formaldehyde to formate can be carried out by the oxygen-sensitive tungsten-dependent aldehyde ferredoxin oxidoreductase (AFOR) which is present in all MAGs except those obtained from Tengchong hot springs (DRTY). In this way, Brockarchaeota can potentially play a key role in controlling formaldehyde consumption and therefore maintaining viability of the microbial community in geothermally active environments.

### Formate, methanol, and trimethylamine assimilation

**Fig. 4:**
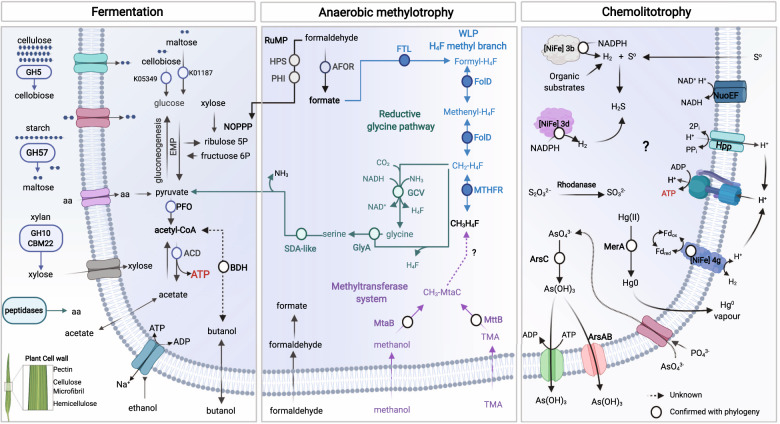
Overview of metabolic capabilities among distinct lineages within the Brockarchaeota phylum. Fermentation panel: Brockarchaeota can breakdown and assimilate of high molecular-weight plant-derived polysaccharides via fermentation and produce ATP by substrate-level phosphorylation via concerted action of PFO (present in all except QC4_43 and JZ-2.136), and ACD, present in hot spring genomes (GD2_1, JZ-1.89, DRTY-6.80, JZ-1.89, QC4_43, QZM_A2, QZM_A3). Acetate can also be assimilated back to acetyl-CoA by an acetyl-CoA synthetase (ACS). Anaerobic methylotrophy panel: convergent pathways for formaldehyde and methylated compound assimilation also exist in Brockarchaeota. The RuMP (shown in black), reductive glycine pathway (rGlyP, shown in green), tetrahydrofolate methyl branch (H_4_F-methyl, shown in blue), and methyltransferase system (shown in purple) that can lead to biomass production and energy conservation. An undescribed protein may be involved in the transfer of C1 moiety from the corrinoid protein to tetrahydrofolate (dashed purple arrows and be metabolized via H_4_F methyl branch of the WLP (blue arrows). Chemolithotrophy panel: brockarchaeota found in hot springs may increase their energy yield from the oxidation of geothermally abundant compounds such as mercury (Hg), arsenate (AsO_4_^3-^), hydrogen (H_2_), and elemental sulfur (S°). Abbreviations. Pathways: EMP Embden-Meyerhof-Parnas, NOPPP Non-Oxidative Pentoses Phosphate Pathway, WLP Wood–Ljungdahl pathway. Enzymes: PFO pyruvate ferredoxin oxidoreductase, and ACD acetate-CoA ligase (ADP-forming), PFL pyruvate formate lyase, ACS acetyl-CoA synthetase, AFOR tungsten-dependent aldehyde ferredoxin oxidoreductase, BDH butanol dehydrogenase (FTL) tetrahydrofolate (H_4_F) ligase, FolD methenyl-THF cyclohydrolase/methylene-THF dehydrogenase, methylene-THF reductase MTHFR (methyltransferase system (MT), GlyA, serine hydroxymethyltransferase (GlyA), SDA-like serine deaminase-like protein, GCS glycine-cleavage system. Compounds: TMA trimethylamine. Dashed lines indicate potential novel enzymatic steps. White circles indicate enzymatic steps confirmed with phylogeny (more details can be found in Supplementary Figure 3). Created with BioRender.com.

In Brockarchaeota formate, derived by the oxidation of formaldehyde, or obtained from their geothermally surroundings^33^, can be further assimilated by two pathways for formate assimilation: the H_4_F-WLP, and the rGlyP. Brockarchaeota code for three key enzymes for the H_4_F-WL including formate-tetrahydrofolate ligase FTL (present in DRTY735_44, JZ-1.89, QC4_43_77, QZM_A3_48), methylene-H_4_F reductase (NADPH) MTHFR (DRTY-6.200, JZ-2.136 and QZM-A3_48), and methylenetetrahydrofolate dehydrogenase (NADP + )/methenyltetrahydrofolate cyclohydrolase FolD (QZM_A2, JZ-1.89, GD2, JZ_2.136, JZZ.4, B27_69, DRTY-6-80). This suggests that formate can be fixed to formyl-H_4_F, and then reduced to generate the active intermediate methylene-H_4_F that can be metabolized via rGlyP. The main component of the rGlyP is the glycine cleavage system (GCS), that catalyzes the reversible cleavage of glycine to CO_2_, CH_2_-H_4_F, and ammonia (NH_3_). The GCS is composed of glycine dehydrogenase (both subunits GcvPA and GcvPB present in B48_G17 and JZ-2.136), aminomethyltransferase (GcvT, present in JZ-2.136), lipoate-binding protein (GcvH, present in B48_G17 and JZ-2.136); Lpd, dihydrolipoyl dehydrogenase (present in DRTY735_44 and DRTY-1.18). This suggests that in Brockarchaeota, like other anaerobes, the GCS operates in reductive direction by condensing the C1 moiety of CH_2_-H_4_F with CO_2_ and ammonia to produce glycine. Glycine can react with CH_2_-H_4_F to produce serine which can be deaminated to pyruvate by serine-dehydratase-like enzyme which the hot spring genotypes encode (including QZM_A248_33, QZM_A348_16, DRTY7.37, QC448_22, DRTY-6.80, DRTY-1.18, DRTY-6.200, and DRTY735_44). Then pyruvate can be further oxidized to acetate and produce ATP at substrate level phosphorylation for energy conservation and biomass production (see Fig. 4).

### An unknown pathway for butanol metabolism

A phylogenetic analysis of alcohol dehydrogenases from the hot spring genomes revealed that they encode a butanol dehydrogenase BDH (Supplementary Fig. 4) that may catalyze the reversible conversion of butyraldehyde to butanol. Brockarchaeota BDH’s are homologues to sequences from obligately anaerobic, thermophilic bacteria that can degrade complex plant saccharides such as xylan (i.e., *Caldicoprobacter oshimai*^34^ and *Hungateiclostridium thermocellum*^35^) or cellulose (*Hungateiclostridium alkalicellulosi)*. To investigate if Brockarchaeota can oxidize or produce butanol, we searched for genes involved in production of butanol in two model organisms; *Clostridium acetobutylicum*^36,37^ which is one of the few organisms that produces butanol as a fermentation product, and *Saccharomyces cerevisiae*^38^ involved in butanol and isopropanol production. We found that Brockarchaeota genomes lack the key enzymes involved in the fermentation of pyruvate to butanol (butanal dehydrogenase, butyryl-CoA dehydrogenase, enoyl-CoA dehydratase, 3-hydroxyacyl-CoA dehydrogenase). However, most of the genomes encode a putative aldehyde dehydrogenase that could convert butyraldehyde to butyric acid. Also, we found a putative enoyl-CoA hydratase/isomerase protein that is coded by one bin (JZ-1.89), which could be involved in further converting butyric acid to acetyl-CoA. Our results suggest an alternative pathway for butanol oxidation that still remains unresolved (Supplementary Fig. 5).

### Utilization of extracellular organic carbon and detrital proteins

Brockarchaeota may be able to degrade a variety of organic carbon compounds. They may utilize hexoses via Embden-Meyerhof-Parnas (EMP) pathway (Supplementary Fig. 6) and pentoses (xylose isomerase XylA and xylulose kinase XylB) via the isomerase pathway (Supplementary Fig. 7). These enzymes were previously only found in bacterial thermophiles and halophilic archaea that ferment complex compounds and degrade xylose suggesting a similar physiology in Brockarchaeota^39^. Once transported into the cell, carbon complex compounds could enter the central metabolism and be converted to acetate and H_2_ via acetogenic fermentation. The ATP conserving step for sugar or pyruvate fermentation to acetate could be catalyzed by acetate-CoA ligase in the hot spring genomes. Acetate can also be converted to acetyl-CoA by acetyl-CoA synthetase (ACS), thus acetate might be a source of carbon and energy in the absence of other substrates in hot spring Brockarchaeota. The presence of pyruvate ferredoxin oxidoreductase (PFO) that couples pyruvate oxidation to H_2_ production, generating acetyl-CoA, could support fermentative metabolism via degradation of either acetate, pyruvate, hexoses, or pentoses. Brockarchaeota genomes code a wide repertoire of ATPases such as the plasma-membrane proton-efflux P-type ATPase (only present in the hot spring genomes), Zn^2+/^Cd^2+-^exporting ATPase (present in DRTY7.37), and the V/A-type H + /Na + -transporting ATPase (in most of the genomes). The existence of ATPase in Brockarchaeota suggests that members of these genotypes have the additional ability to couple acetogenic fermentation to membrane potential generation.

**Fig. 5:**
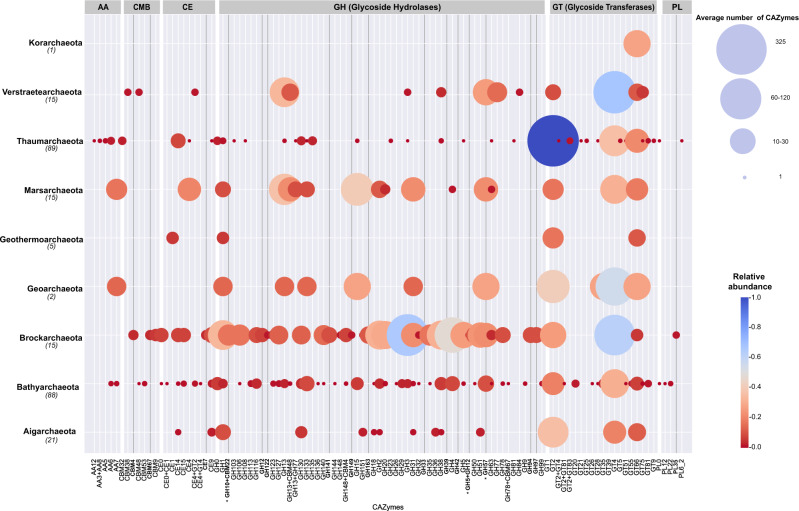
Carbohydrate-active enzymes (CAZymes) encoded by genomes belonging to the TACK superphylum including Brockarchaeota. The total number of CAZymes per phylum was normalized by the total number of genomes described for each phylum shown in parenthesis. Auxiliary activities (AA), carbohydrate-binding module (CBM), carbohydrate esterases (CE), glycoside-hydrolases (GH), glycoside transferases (GT), polysaccharide lyases (PL). Unique Brockarchaeota CAZYmes are shown in darker lines. Extracellular CAZYmes are shown in asterisks described in Supplementary Data 12 sheet 3.

To complement their ability to degrade xylose, Brockarchaeota also contain a relatively high number of carbohydrate-active enzymes (average of 27 CAZYmes per genome) which is 3 times what has been observed in other TACK archaea (Supplementary Data 12 and Fig. 5). Ten of the 15 Brockarchaeota genomes have genes with similarity to α-L-fucosidase involved in the degradation of xyloglucan, which is the major component of hemicellulose in plant-cell walls^40^. All the hot spring genomes encode GH3 family proteins which may be involved in biomass degradation, but these proteins play other roles in cell wall remodeling, energy metabolism, and pathogen defense^41^. The hot spring genotypes contain a wider repertoire of CAZymes than the deep-sea GB genomes. Among these are four predicted to be extracellular glycoside hydrolases, which are involved in the breakdown of high molecular-weight plant-derived polysaccharides, primarily xylanes, cellulose, and starch. Comparison of the CAZYmes across the TACK superphylum revealed that 17 extracellular enzymes, including those for the degradation xylanes, are unique to Brockarchaeota (Supplementary Data 12). The diversity and abundance of CAZYmes in members of the TACK superphylum highlights that despite the low number of sequenced Brockarchaeota genomes (15 described in this study), compared to Thaumarchaeota (89), they contain a greater variety and number than their closest relatives. Brockarchaeota also encode a wide range of peptidases. With exception of some bins (B48_G17, DRTY-6.200, DRTY7_35_44, JZ-1.89, JZZ.4, and QC4_43), they all encode potential extracellular peptidases indicating a potential role of Brockarchaeota in protein remineralization. Many of the are so unique that is was not possible to identify their specific substrates (Supplementary Data 13).

### Mercury, sulfur, arsenate, and hydrogen chemolithotrophy

Geothermal ecosystems such as shallow, and deep-sea vents, volcanoes, geysers, hot springs, and fumaroles are natural sources of mercury (Hg)^42^. Hg resisting microorganisms are known to be enriched in deep-sea hydrothermal vents and in terrestrial geothermal springs. Three hot springs Brockarchaeota genomes (DRTY735_44, DRTY-1.18 and DRTY7.37) encode mercuric reductase (MerA), the central enzyme in the microbial mercury detoxification system. MerA transforms the extremely toxic Hg (II) to metallic Hg(0), being potentially involved in mercury detoxification^42^. Brockarchaeota MerA and closely related proteins were used to generate a mercury reductase phylogeny (Supplementary Fig. 8), indicating that Brockarchaeota possess a previously uncharacterized class of MerA, which are related to other archaea (Crenarchaeota, Methanomicrobia, DPANN, and Asgard).

Interestingly, Brockarchaeota also code the arsenic detoxification system that acts by decreasing the intracellular arsenic concentration by pumping out arsenate that enters the cell, thus preventing the metals from accumulating and denaturing proteins^43^. The intracellular dependent arsenate reductase (ArsC, K03741) that catalyzes the reduction of arsenate AsO_4_^3−^ to arsenite As(OH)_3_ (Fig. 4), is present in most hot spring genomes (Supplementary Data 6). Phylogeny of Brockarchaeota ArsC (Supplementary Fig. 9) indicates that they belong to a deep uncharacterized branch of Thioredoxin-coupled clade, that has been mainly described in Firmicutes^44^. In agreement with the geothermal origin of Brockarchaeota genomes, homologous ArsC sequences recovered from geothermally active environments belonging to uncultured Bathyarchaeota or Thaumarchaeota^45,46^, which could potentially be Brockarchaeota, or have a similar arsenate metabolism. The presence of ArsC and the energy-dependent related detoxification proteins, could also indicate that Brockarchaeota in hot spring genomes could use arsenate as terminal electron acceptor, as seen in other bacteria, yet the exact molecular mechanism of this process is unknown^43,47^.

Furthermore, similar to other heterotrophic fermentative hyperthermophilic archaea^48,49^ Brockarchaeota might be able to reduce elemental sulfur during fermentative growth and produce H_2_S due to the presence of [NiFe] Group 3b hydrogenases (Supplementary Fig. 10). During carbohydrate fermentation in the absence of sulfur, [NiFe] Group 3b hydrogenase can catalyze the production of H_2_ with NADPH or NAD(P)H as the electron donor. However, in the presence of sulfur, Brockarchaeota might have the ability to reduce sulfur using H_2_ or organic substrates as electron donors, a widespread physiology in hyperthermophilic archaea living in geothermally active environments (volcanic habitats, hots springs, or marine sediments).

Hydrogen is also abundant in geothermally active systems due to volcanic processes^50^. Brockarchaeota might be able to use 3b [NiFe]-hydrogenases for H_2_ oxidation with NADP + or NAD(P) + as an electron acceptor^51^. The hot spring genomes also encode oxygen-tolerant group 3d [NiFe]-hydrogenases, which may allow them to transfer electrons between NAD(P)H and H_2_ depending on the availability of electron acceptors. Group 3d [NiFe]-hydrogenases are abundant in metagenomes from hot springs where microbial communities are relatively stable despite partial pressure of oxygen fluctuations^52^. Group [NiFe] 3b hydrogenases may also make it possible for these archaea to reduce elemental sulfur to H_2_S during fermentative growth. During carbohydrate fermentation in the absence of sulfur, Group 3b [NiFe]-hydrogenases might catalyze the production of H_2_ with NADPH or NAD(P)H as the electron donor. Therefore, Brockarchaeota might have the ability to reduce sulfur, using H_2_ or organic substrates as electron donors, which is common in hyperthermophilic archaea living in geothermally active environments^53^.

## Discussion

**Fig. 6:**
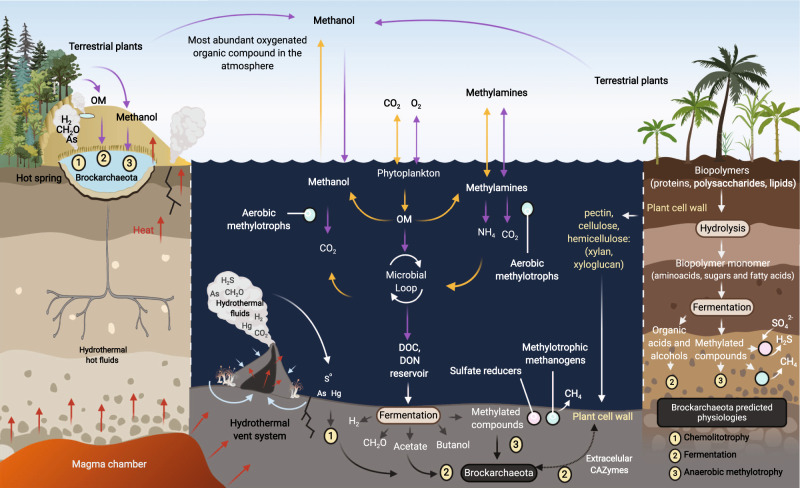
A model of the biogeochemical roles of Brockarchaeota in the anaerobic carbon cycle. C1 and methylated compounds, such as methanol or methylamines, are utilized biologically as carbon and energy sources in the ocean and deep-sea sediments resulting in a considerable carbon reservoir. The biodegradation of organic carbon in the water column and subsurface is a source of these compounds. The utilization of methyl compounds as precursors in methane synthesis is confined to a small group of methylotrophic methanogens (i.e., Verstraetearchaeota). The only described anaerobic methylotrophs include members of methanogenic archaea, acetogenic bacteria, and sulfate-reducing bacteria. These organisms compete for these compounds geochemically produced in anoxic settings. Brockarchaeota may recycle methanol and methylamines in anoxic environments without methane formation and may be sequestered in deep sea sediments and hot springs. Orange and purple arrows represent sources and sinks, respectively. Organic Matter (OM) includes dissolved and particulate organic matter feeding the microbial loop (adapted from Evans et al., 2019 and Zhuang et al., 2018).

Brockarchaeota gene content indicates they are facultative or obligate anaerobic fermentative organisms that produce acetate, CO_2_, and H_2_ as byproducts (see Supplementary Information for details). Some Brockarchaeota have unique pathways for non-methanogenic methylotrophy. This puts them a unique ecological position in nature, where they degrade abundant methylamines in anoxic environments without the production of methane (Fig. 6). Brockarchaeota are also able to degrade complex carbon compounds such as xylan. Xylans are a major structural polysaccharide in plant cells, and is the second most abundant polysaccharide in nature, accounting for approximately one-third of all renewable organic carbon on Earth after cellulose^54,55^. This suggests that Brockarchaeota are players in organic matter degradation in geothermally active environments. Interestingly, detrital proteins can be used as a substrate by Brockarchaeota, indicating potential role in protein remineralization in geothermally active environments.

The protein repertoire of GB and hot spring genomes have some important distinctions that reflect different anaerobic metabolisms. GB genomes appear to be obligately fermenting organisms that rely mostly on substrate-level phosphorylation since they lack all the complexes for the respiratory chain with exception of the ATPase. In contrast, hot spring genomes appear to have mechanisms to increase their ATP yield including the use of geothermally derived inorganic substrates as possible terminal electron acceptors such as mercury (Hg), arsenic (As), and hydrogen (H_2_). Deep-sea hydrothermal vents, hot springs, and fumaroles are natural sources of Hg^42^, H_2_^52^, arsenic^56^, and sulfur^57^.

The discovery of Brockarchaeota genomes from sediments around the world, overlooked by conventional rRNA gene diversity approaches, highlights the need for further exploration of subsurface microbial communities. Although they are relatively low in abundance in the communities described here, the addition of these genomes to public databases, will enhance their detection in future environmental studies, like other recently described novel archaeal lineages^1,58^. A lack of recognition of their existence prior to this limited our ability to fully describe sediment community structure and function. Given their broad distribution, and versatile carbon metabolism, they are likely key players in global carbon cycling. However, this first description is limited to genomic characterization, thus culturing or in activity measurements are needed to confirm their physiological activities^59^. Overall, the description of this new phylum enhances our understanding of biodiversity of archaea and suggests they are mediating unique roles in anoxic carbon cycling.

## Methods

### Metagenomic assembly and binning

Two MAGs (B48_G17 and B27_G9) were obtained from Guaymas Basin sediments (Gulf of California; 27°N0.388, 111°W24.560) and were obtained as part of a larger study of these hydrothermal marine sediments^25^. Both samples were collected from the same location but G9 was sampled from 0–3 cm and G17 from 12–15 cm depth. The sediment cores from which these two MAGs were binned from were collected during Alvin dive 4571_4 in 2009 using polycarbonate cores (45–60 cm in length, 6.25 cm interior diameter), subsampled into cm layers under N_2_ gas in the ship’s laboratory and immediately frozen at −80**°**C. Details on the sampling site and metagenomic sequencing effort is provided in Dombrowski et al.^25^. Briefly, total DNA from ≥10 g of sediment from each sample was extracted using the MoBio PowerMax soil kit using the manufacturer’s instructions and adjusted to a final concentration of 10 ng/µl of each sample (using a total amount of 100 ng). Libraries for paired-end Illumina (HiSeq–2500 1TB) sequencing were prepared by the Joint Genome Institute (JGI). Sequencing was performed on an Illumina HiSeq 2500 machine using the paired-end 2 × 125 bp run-type mode. All runs combined provided a total of ~280 gigabases of sequencing data. Quality control and sequence assembly were performed by JGI. Briefly, sequences were trimmed and screened for low-quality sequences using bbtools (https://jgi.doe.gov/data-and-tools/bbtools/) and assembled using megahit v1.0.6 using the following options: --k-list23,43,63,83,103,123. For further binning, only scaffolds ≥2000 bps were included.

Metagenomic binning was performed on individual assemblies using the binning tools ESOM, Anvi’o (v2.2.2)^60^ and Metabat (v1)^61^. ESOM bins were extracted using getClassFasta.pl and the command -loyal 51. Anvi’o was run with default parameters and metabat was run using the following settings: --minProb 75 --minContig 2000 --minContigByCorr 2000. Results from the three different binning tools were combined using DAS Tool (version 1.0) as follows: DAS_Tool.sh -i Anvio_contig_list.tsv, Metabat_contig_list.tsv, ESOM_contig_list.tsv -l Anvio, Metabat, ESOM -c scaffolds.fasta --write_bins 1.

Eight MAGs (DRTY-1.18, DRTY-6.80, DRTY-6.200, DRTY7.37, JZ-1.89, JZ-2.136, JZZ_4, and DRTY7) were recovered from hot springs in Yunnan, China collected in January of 2016 and May of 2017 in several hot springs (Supporting Data 1). Five additional MAGs (QC4_43, QC4_48, GD2_1_47_42, QZM_A2, QZM_A3) were reconstructed from hot springs in Tibet in August of 2016. Sequencing was done on an Illumina HiSeq4000 (Beijing Novogene Bioinformatics Technology Co., Ltd). These samples were assembled using metaSPADES (version 3.9.1), with a k-mer set of “21, 33, 55, 77, 99, 127”. For each sample only scaffolds larger than 2500 bp were binned using MetaBAT (v.2.12.1) with default parameters, considering both tetranucleotide frequencies (TNF) and scaffold coverage information. The scaffolds from the obtained bins and the unbinned scaffolds were visualized using ESOM with a minimum length of 2500 bp and maximum length of 5000 bp as previously described^62^ and the bins were modified by removing any out-of-range scaffolds (indicated by sequence points) or adding any unbinned scaffolds using ESOM related scripts^37^. MAGs from Tibet hot springs with scaffolds ≥1000 bp were uploaded to ggKbase (http://ggkbase.berkeley.edu/), and the bins from ESOM analyses were evaluated and modified manually at ggKbase based on GC content, coverage, and taxonomic information of scaffolds. MAGs from Tengchong hot springs were reassembled using SPAdes (version3.9.1) under the “careful” mode with the same k-mers. During this step, the reads used for the assemblies were recruited by mapping clean reads to the curated genome bins using BBmap (v35.85; http://sourceforge.net/projects/bbmap/). The accuracy of all the MAGs was evaluated by calculating the percentage of completeness and gene duplications using CheckM lineage_wf (v1.0.5).

### Phylogenetic analyses

A phylogenetic tree was generated as recently described in ref. ^1^. Briefly, 37 conserved marker proteins were extracted using phylosift^63^, in a genomic dataset containing 3549 archaeal genomes including Brockarchaeota, and 40 bacterial genomes. An alignment of the proteins extracted from a total of 3599 genomes was generated using MAFFT v7.450^64^ (algorithm autoselection) with a BLOSUM62 scoring and contains 4962 characters after masking gaps present in at least 50% of the taxa. A tree was constructed with IQtree^65^ v1.6.11 with a best fit LG + F + R10 model selected using the Bayesian Information Criterion (BIC) and bootstraps are based on 1,000 replicated trees. Command-line options -bb 1000 -bnni The bacterial genomes were used as an outgroup. The 16S rRNA sequences were extracted from Brockarchaeota genomes using Barrnap v.09 (https://github.com/Victorian-Bioinformatics-Consortium/barrnap) with the following parameters: --kingdom arc --lencutoff 0.2 --reject 0.3 –evalue. The obtained sequences (Supplementary Data 7). were used for a 16S rRNA gene phylogeny that included sequences derived from metagenomic surveys (NCBI accession EU924237, KX213943, and KX213897) and the IMG database. We used the 16S rRNA genes from the MAGs to search these databases to identify additional Brockarchaeota genes. The rRNA phylogeny was generated using RAxML within the ARB software package (v. 2.5b). using default parameters.

### Metabolic predictions

Gene predictions for individual genomes were performed using Prodigal^66^ (V2.6.2, default settings). Predicted genes of individual genomes were further characterized using a combination of several databases: KofamKOALA^67^, Interproscan v5.31.70^68^, HydDB^69^, dbCAN2^70^, MEBS^71^, METABOLIC^72^, and MEROPS v12.1^73^. For KofamKOALA only hits above the predefined threshold for individual KOs were selected. Hydrogenases were extracted using the reference database described in Greening et al. and Søndergaard et al.^52,69^ where there was conflict, the protein was manually reanalyzed using BLAST against non-redundant protein database, and genomic organization and annotation was confirmed using a web-based tool Operon Mapper^74^. The detected hydrogenases were used to generate a phylogenetic tree as previously described in Seitz et al.^7^. Hits for key metabolic marker genes were verified across different databases KofamKOALA, PFAMv31 and TIGRFAMs and HydDB and were further verified using BLASTP using the NCBI web server tool. Genes encoding for carbohydrate degradation enzymes described in the carbohydrate-active enzymes (CAZYmes) database^75^ were identified by only retaining hits recovered by ≥2 tools. Protein localization of the selected CAZYmes and peptidases was determined with the command line version of Psort v3.0^76^ using the options -a and -terse for archaeal genomes in tabular format files. Finally, the presence of specific protein families was obtained with MEBS. The annotation was performed in a genomic dataset of 250 publicly available TACK genomes (Supplementary Data 3) that were also used for the CAZYmes annotation.

### Methyl coenzyme M reductase screening

The *mcrA* gene was identified using GraftM v0.10.2^77^ across metagenome assemblies where Brockarchaeota genomes from hot springs were detected^78^. The *mcrA*-containing scaffolds with sequence length <2.5 Kbp were discarded since scaffolds with short length were not used during the genome binning step. The taxonomic information of the corresponding bins which contain *mcrA* genes were determined using either GTDBtk v0.3.2^79^ or phylogenetic placement (as reported in Supplementary Data 11). The *mcrABGCD* genes were identified in metagenome assemblies from deep-sea assemblies previously described^25^ (Guay17 and Guay9; IMG genome ID 3300014887 and 3300013103, respectively).

### Relative abundance of Brockarchaeota in communities

The relative abundance of the MAGs from deep sea samples was obtained from Supplementary Data 3 in Dombrowski et al. ^25^. Only samples G9 and G7 are shown, and the data is sorted according to the relative abundance of those corresponding samples. GB MAGs include a post publication manual refinement in the taxonomy according to the recent archaea tree of life^1^.The relative abundance of MAGs from Tenghchong samples was computed using the bin_abundance.py script (https://github.com/valdeanda/MetaGaia). For each MAG, total length of mapped reads for individual scaffolds (mapped reads using BWA algorithm) is summed up and the total is then divided by the MAG size in bp. This number is then divided by the total number of reads to obtain the relative abundance. The final relative abundance is multiply it by 100000000 for readability purposes. For the Tibet samples, the genome bins obtained for a given sample, the sequencing coverage was determined by read mapping using Bowtie2 and coverage calculation using the jgi_summarize_bam_contig_depths script from MetaBAT^61^. The relative abundance of a given genome bin was calculated as its sequencing coverage divided by the total sequencing coverage of all genome bins in the corresponding sample (Tengchong samples as previously described)^6^.

### Reporting summary

Further information on research design is available in the Nature Research Reporting Summary linked to this article.

## Supplementary information

Supplementary Information

Description of Additional Supplementary Files

Supplementary Data 1

Supplementary Data 2

Supplementary Data 3

Supplementary Data 4

Supplementary Data 5

Supplementary Data 6

Supplementary Data 7

Supplementary Data 8

Supplementary Data 9

Supplementary Data 10

Supplementary Data 11

Supplementary Data 12

Supplementary Data 13

Reporting Summary

## Data Availability

The final assembled and annotated genomic sequences of Brockarchaeota from deep sea sediments (B27_G9 and B48_G17) have been deposited in NCBI under BioProject ID PRJNA362212: BioSample id SAMN09215183 and SAMN09214986, respectively. Sequence data and sample information of Brockarchaeota from hot springs are available at NCBI under Bio Project ID PRJNA544494. All the NCBI accession numbers for the MAGs described in this study are provided in Supplementary Data 1. Raw data for Figs. 1, 2 are provided as Supplementary Data 3 and 7 respectively. Any other datasets generated and/or analyzed during the current study are available from the corresponding authors on request.

## Code availability

All custom scripts have been made available at https://github.com/valdeanda/Useful_scripts. The protocol to compute MAGs relative abundance is available at MetaGaia (https://github.com/valdeanda/MetaGaia) bin_abundance.py script.
