# Supplementary material for: Brockarchaeota, a novel archaeal phylum with unique and versatile carbon cycling pathways
Source: Nat Commun. 2021 Apr 23;12:2404. doi: 10.1038/s41467-021-22736-6 (PMC8065059; doi:10.1038/s41467-021-22736-6)
Supplement: Supplementary file 1 — Supplementary Information [file 41467_2021_22736_MOESM1_ESM.pdf]

Supplementary Information

Brockarchaeota, a novel archaeal phylum with unique and versatile carbon cycling pathways

De Anda et al.

Table of Contents

Supplementary Discussion 2

Genomic and environmental characteristics of Brockarchaeota spp. 2

Central metabolism 2

Glycolysis/Gluconeogenesis: reminiscent of early life and sugar flexibility 2
Pentose phosphate pathway (PPP) and ribulose monophosphate (RuMP) pathway 3
Formate assimilation 4
Aerobic methylotrophy 6
Potential new anaerobic methylotrophic pathway 6
Degradation of complex carbohydrates 7
Degradation of detrital proteins 8
Catabolism of pentoses: xylose degradation. 8
Fermentation 9
Butanol degradation 10

Detoxification or energy conservation mechanisms 10

Arsenate. 10
Mercury. 11
Sulfur cycling 11
Hydrogen utilization 12

Supplementary Figures 13

Supplementary Discussion

Genomic and environmental characteristics of Brockarchaeota spp.
Brockarchaeota metagenomic assembled genomes (MAGs) are estimated to be 67-92% complete based on checkM. The genome sizes of these MAGs range from 0.78 to 2.32 Mbp (average 1.47 Mbp) (Table 1). Two MAGs from Guaymas Basin (GB; B48_G17 and B27_G9, temperature 33.6 and 10.4°C respectively) were originally referred to as "GB-AP1" in Dombrowski et al., 2019 Although the GB genomes were obtained from a lower temperature environment compared with the Chinese MAGs (obtained from hots springs up to 86.5°C) (See Supplementary Data 1) metagenomes from GB, were collected in close proximity to hydrothermal circulation and experience increases in temperature due to the dynamic nature of these sediments. Although these environments are primarily anoxic, the hot spring (QC4) did have measurable oxygen (DO; 1.85mg/L) in agreement with a larger redox potential (59 mV) in this site, compared with more anoxic and reducing conditions found in the GD2_1 and QZM_A2 (-191 and -160 mV respectively). Low sulfide concentrations (up to 13.16 mg/L in B48_G17 from GB), and high sulfate were characteristic of GB sediments (up to 210 mg/L G27_G9) (See Supplementary Data 1). Therefore, it seems that Brockarchaeota genomes thrive in geothermal ecosystems, preferably at high temperatures at the expense of inorganic and organic compounds under anoxic conditions. Consistently, several Brockarchaeota spp. encode reverse gyrase (Supplementary Data 6) essential for life under hyper thermophilic conditions. However as other hyperthermophiles, Brockarchaeota genomes were recovered from anoxic environments ranging from 10 up to 86.5°C.

Central metabolism

Glycolysis/Gluconeogenesis: reminiscent of early life and sugar flexibility
Brockarchaeota MAGs encode most of the steps of glycolysis via Embden-Meyerhof-Parnas (EMP) pathway as well as gluconeogenesis (Supplementary Figure 6). However, unlike most sugar-utilizing (hyper)thermophilic Archaea analyzed so far, where the glyceraldehyde 3-phosphate (GAP) oxidation is carried out in one step without 1-3BPG formation and, thus, without coupling of the oxidation of GAP to 3PG with the synthesis of ATP via substrate-level phosphorylation[1], Brockarchaeota MAGs can potentially use two different strategies.

(a) The hot spring genomes encode the glyceraldehyde-3-phosphate dehydrogenase GAPDH [EC: 1.2.1.12]) and phosphoglycerate kinase PGK [EC:2.7.2.3]. The presence of these enzymes indicate that they could couple the reversible Pi - dependent oxidation of GAP with the concomitant generation of ATP via substrate-level phosphorylation, with 1,3BPG as an intermediate[1]. While a GAPDH/PGK might be involved in GAP oxidation in glucose catabolism in hot spring genomes.

(b) The deep-sea GB genome B48_G17 bin was found to encode the NAD(P)+-dependent glyceraldehyde-3-phosphate dehydrogenase (EC 1.2.1.59) that might be involved in gluconeogenesis/glycolysis.

Brockarchaeota genomes encode for most glycolytic enzymes including ATP-dependent phosphofructokinase/diphosphate-dependent phosphofructokinase (K21071), 6-

phosphofructokinase (K00850); only present in JZ-2.136) pyruvate kinase (K00873) and both GAPH/PGK and GPDHII, which might reflect their ability to degrade sugars, consistent with their broad spectrum of Carbohydrate-Active Enzymes (CAZymes) (see discussion below). The phosphomannomutase/phosphoglucomutase (pgm) a key enzyme that serves as branch point in glucose metabolism for energy synthesis and cell surface construction providing substrates for the PPP pathway or the synthesis of polysaccharides from glucose-6-phosphate or mannose-6-phosphate[3] was present in most of the Brockarchaeota genomes (Supplementary Figure 6)

Another difference is the presence of the heat-stabile fructose 1,6-bisphosphate aldolase/phosphatase [EC:4.1.2.13/3.1.3.11] (Fba, (K01622) present in GB genome B48_G17, a key ancestral gluconeogenic enzyme that acts as a unidirectional gluconeogenic pathway under conditions where the carbon flux does not need to be turned to sugar degradation[2]. The same reaction can be performed by fructose-bisphosphate aldolase/ 2-amino-3,7-dideoxy-D-threo-hept-6-ulosonate synthase [EC:4.1.2.13/2.2.1.10] (K16306) detected in the hot spring genomes JZ-2.136 and JZZ-4. Also, Brockarchaeota from hot springs encode Fructose-1,6-bisphosphate aldolase (FBPA), class II (EC 4.1.2.13, K01624). Since this key enzyme for gluconeogenesis and glycolysis is widely distributed in bacteria and fungi, and rarely found in archaea, it has been suggested that it might be a HGT event from bacteria to haloarchaea[1] where they have been found.

We found the key enzyme of the reductive TCA cycle 2-oxoglutarate synthase in Brockarchaeota. One bin from the deep sea (B48-G17) encodes all the subunits (KorABDG), while the other Brockarchaeota MAGs encode one or two subunits (Supplementary Data 6). B48-G17 also encodes both subunits of the fumarate hydratase enzyme FumAB (K01677, K01678) indicating a production of organic molecules for biosynthesis via reductive TCA cycle in Brockarchaeota from the deep-sea. However, more deep-sea Brockarchaeota genomes will be necessary to confirm whether they can use the incomplete reductive TCA cycle for biosynthesis or intermediates as has been proposed in some members of methanogenic archaea[3].

Pentose phosphate pathway (PPP) and ribulose monophosphate (RuMP) pathway

Although the complete Non-Oxidative Pentoses Phosphate Pathway (NOPPP) is rare in Archaea, homologous of all four key enzymes; ribose-5-phosphate isomerase (RPI) ribose-5-phosphate-3-epimerase (RPE), transketolase (TK); transaldolase (TA) are present in Brockarchaeota. This suggests that Brockarchaeota MAGs are able to synthesize key biosynthetic intermediates (R5P and E4P) via the reverse reactions of the NOPPP from F6P and glyceraldehyde 3-phosphate. Interestingly, Brockarchaeota MAGs (B48_G17, DRTY7_35_44, DRT-1.18, JZ-2.136, DRTY7.37) encode both key enzymes of the ribulose monophosphate (RuMP) pathway: 3-hexulose-6-phosphate synthase (HPS) and 6-phospho-3-hexuloisomerase (PHI). The Brockarchaeota MAG DRTY-6.200 only encodes PHI. The rest of the MAGs lack both enzymes (See Pentoses Phosphate Pathway and Ribulose Monophosphate pathway in Supplementary Figure 7).

The RuMP pathway was originally found in methylotrophic bacteria that are able to use C1 compounds as a sole source of carbon and energy, however, it is currently recognized as a widespread prokaryotic pathway for formaldehyde fixation and detoxification (Orita et al., 2006). Formaldehyde was an essential building block to synthesize sugars on early Earth and is ubiquitous in nature, produced through the degradation of compounds containing methyl or methoxyl groups, e.g., lignin and pectin[4]. In geothermally active systems such as in hydrothermal vents and hots springs, formaldehyde is expected to be present since C1 compounds are found in a variety of redox levels (i.e CH_4, CO_2, CO), and could be produced by autotrophic microorganisms found to

be present in geothermally active environments such as *Metallosphaera yellowstonensis MK1*[5]. The RuMP pathway functions as a highly efficient system for trapping free formaldehyde at relatively low concentrations. The presence of HPS and PHI in Brockarchaeota genomes suggests that formaldehyde can be fixed and detoxified via the RuMP pathway. Brockarchaeota can potentially play a key role in controlling formaldehyde consumption and therefore maintaining viability of the microbial community in geothermally active environments.

Further processing of formaldehyde to formate can be carried out by the oxygen-sensitive tungsten-dependent aldehyde ferredoxin oxidoreductase (AFOR) (K03738) which is present in all MAGs except those obtained from Tengchong hot springs (DRTY) (Supplementary Data 6 Raw sheet, and Supplementary Figure 3). Since AFOR catalyzes the oxidation of aldehydes (including formaldehyde) to their corresponding acids, it is likely that in Brockarchaeota, could be involved in the oxidation of formaldehyde to formate that can be further assimilated into central metabolism (See more details below).

Formate assimilation

The only known formate assimilation pathways are the Wood–Ljungdahl pathway (WL), the serine pathway, and the reductive glycine pathways[6] (See black arrows Supplementary Figure 3) There are two main strategies by which microbes can grow on formate as the sole carbon source. The first one involves being oxidized completely to CO_2, and the second one involves the condensation of formate with another metabolic intermediate[6]. Based on detailed comparative gene contents it appears that Brockarchaeota can assimilate formate using two convergent routes by either using the tetrahydrofolate (H_4F) methyl branch of the WL pathway or via the reductive glycine pathway.

Here are the details about why we think this is the case:

1. **Serine cycle is not likely to be operating in Brockarchaeota**. In Brockarchaeota, the enzyme malate dehydrogenase (oxaloacetate-decarboxylating) was found to be only present in one deep sea MAG (B27_G9). This and the absence of other key serine cycle enzymes (to regenerate glyoxylate and malate) suggests that the ATP-costly serine cycle is not being used as a potential formate assimilation pathway (See supporting Figure 2).
2. **Lack of the canonical carbonyl branch of the WLP.** In contrast to acetogenic bacteria, where the methylenetetrahydrofolate (CH_2-H_4F) is reduced to methyltetrahydrofolate (CH_3-H_4F), and then transfers its methyl moiety to a corrinoid iron−sulfur protein to react with CO_2 and CoA within the CO-dehydrogenase−acetyl-CoA-synthase complex (See Supplementary Figure 3). Brockarchaeota lacks the later enzyme (CO-dehydrogenase−acetyl-CoA-synthase complex) indicating that the canonical acetogenic model is not likely to be operating in these archaea.
3. **Key enzymes of the H_4F methyl branch of the WLP are present in hot spring Brockarchaeota.** The presence of formate-tetrahydrofolate ligase FTL [EC:6.3.4.3] in hot spring MAGs (DRTY735_44, JZ-1.89, QC4_43_77, QZM_A3_48) indicates that formate can be fixed to formyl-H_4F, and then reduced to generate the active intermediate methylene-H_4F. Interestingly, FTL is the only formate-fixing reaction that is known to support microbial growth using formate as sole carbon source, suggesting a key role in formaldehyde and formate metabolism in hot spring genomes. Other two key enzymes of the H_4F methyl WL pathway were found to be present in Brockarchaeota genomes: 1) Methylene-H_4F reductase (NADPH) MetFV [EC:1.5.1.20] (DRTY-6.200, JZ-2.136 and QZM-A3_48 MAGs), which could be involved in electron bifurcation (see below), and 2) methylenetetrahydrofolate dehydrogenase

(NADP+)/methenyltetrahydrofolate cyclohydrolase FolD [EC:1.5.1.5/3.5.4.9] (QZM_A2, JZ-1.89, GD2, JZ_2.136, JZZ.4, B27_69, DRTY-6-80) (See Supplementary Figure 3).

4. **Potential electron-bifurcating mechanism in the H_4F methyl branch.** The only reaction of the WLP that can conserve energy and increase the ATP yields is the reduction methylenetetrahydrofolate (CH_2-H4F) to methyltetrahydrofolate (CH_3-H_4F) by the methylene-H_4F reductase MTHR (MetFV) [EC:1.5.1.20] present in three hot spring MAGs (DRTY-6.200, JZ-2.136 and QZM-A3_48). In acetogenic bacteria, the reduction of CH_3-H_4F with NADH via MetFV, drives the reduction of ferredoxin forming a flavin-based electron bifurcation complex MetFV-HdrABC-MvhD[7,8]. Since in some acetogenic organisms (like *M. thermoacetica*) MetFV forms a genetic and functional complex with HdrABC and MvhD[7], we examined the neighboring genes, and operons of MetFV and no *hdrABC* or *mvhD* genes where found. However, we found that MetFV forms transcriptional unit with a uncharacterized membrane-associated protein/domain (COG2512), ferredoxin (COG1145), and thioredoxin (COG0492) (Supplementary Data 9). Furthermore, the presence of group 3C [NiFe]-hydrogenase in the hot spring genotypes (Supplementary Figure 10) suggests possible role bifurcation electron from H_2 to heterodisulfide and ferredoxin. Additionally, the presence of group 4G [NiFe] hydrogenases in JZ-136 and deep-sea genome B48_G17 suggest that these organisms couple ferredoxin$_{red}$ oxidation to proton reduction leading to a gradient driving ATP synthesis and regenerating H_2 recycled for a probable bifurcating complex. ATP synthesis can be catalyzed by the ATP synthase dependent on the Na^+ and H^+ gradients. The involvement of the 4G [NiFe], 3C [NiFe] and MetFV in Brockarchaeota will require more genomes to confirm these genomic-based molecular hypotheses.

5. **Glycine formation via reductive glycine pathway.** The reductive glycine pathway (rGlyP), has been found in amino acid and purine-degrading bacteria providing an alternative formate-consuming acetogenic route[6]. The rGlyp provides several physiological advantages including dissipate the excess reducing power, support efficient one-carbon and glycine metabolism, provides greater ATP efficiency than the serine cycle, and lower overlap with central metabolism[6]. Recently, It has been demonstrated that the rGlyP, is natural fixation pathway, where CO_2 is first reduced to formate, which is then reduced and condensed with a second CO_2 to generate glycine[6,9]. Although the metabolism of Brockarchaeota does not seem to indicate any autotrophic metabolism (lacks any known enzymes of CO_2 fixation pathways), it encodes key components of the rGLyP including the glycine cleavage system (GCS). The GCS that catalyzes the reversible cleavage of glycine to CO_2, CH_2-H_4F and ammonia, is composed of GcvP, glycine dehydrogenase (both subunits GcvPA and GcvPB present in B48_G17 and JZ-2.136); GcvT, aminomethyltransferase (present in JZ-2.136); GcvH, lipoate-binding protein (present in B48_G17 and JZ-2.136); Lpd, dihydrolipoyl dehydrogenase (present in DRTY735_44 and DRTY-1.18). As in other anaerobic microorganisms, we suggest that Brockarchaeota in GCS operates in reductive capacity by condensing the C1 moiety of CH_2-H_4F with CO_2 and ammonia to produce glycine[9]. In this way, the direct substrate of GCS for reductive carboxylation can be produced in Brockarchaeota genomes via FTL and FolD enzymes (as we explain in detail in points 3 and 4 above) and glycine can be produced via rGlyP. The canonical rGlyP the glycine produced by the reductive action of the GCS, can be metabolized in two possible ways either via oxygen-sensitive selenoenzyme glycine reductase (absent in Brockarchaeota) to reduce glycine to acetyl-phosphate which can be converted to acetate and ATP, or the ATP-efficient route using glycine hydroxymethyltransferase GlyA (present in all but six MAGs (B48_G17; DRTY7_35_44; DRTY-1.18; DRTY-6.200;

DRTY7.37; QC4_48). In the later pathway, glycine reacts with another methylene-THF (or CH_2-H_4F) to produce serine which is can be deaminated to pyruvate by serine-dehydratase-like enzyme which the hot spring genomes appear to encode (including QZM_A248_33, QZM_A348_16, DRTY7.37, QC448_22, DRTY-6.80, DRTY-1.18, DRTY-6.200 and DRTY735_44). Then pyruvate can be further oxidized to acetate and produce ATP at substrate level phosphorylation for energy conservation (See Supplementary Figure 6).

Aerobic methylotrophy

In aerobic methylotrophic bacteria, formaldehyde produced from the oxidation of C1 compounds is assimilated to form intermediates of the central metabolic routes that are subsequently used for biosynthesis of cell material i.e., via serine pathway (See Supplementary Figure 2). Screening for key aerobic methylotrophic genes (See Supplementary Figure 2) indicate that Brockarchaeota lack the main the main enzymatic steps including the PQQ-linked methanol dehydrogenases (MDH) and NAD-linked MDH found in aerobic methylotrophic bacteria. They also lack methane monooxygenases (MMO) found in methanotrophic organisms. Brockarchaeota lacks the methylamine dehydrogenase pathway that oxidizes C1 compounds to formate (Supplementary Figure 2 left side panel) and the essential genes for the alternative pathway that is found in organisms that can grow on C1 compounds and lack methylamine dehydrogenase. This alternative pathway proceeds via N-methyl-L-glutamate synthase complex (MgsABC). Only one Brockarchaeota genome encodes MgsC and but lacks the rest of the subunits for the complex as well as the enzymatic steps for the pathway. Due to the presence of glycine hydroxymethyltransferase (SHMT), is possible that formaldehyde can react with glycine to form L-serine which can be converted to pyruvate via threonine dehydratase [EC 4.3.1.19] and serve as be an entry point to the central metabolism in the form of pyruvate as was explained above.

Potential new anaerobic methylotrophic pathway

Despite the presence of methyltransferase system-related proteins, Brockarchaeota genomes do not possess the common core marker genes specific to methanogenesis including methyl-coenzyme M reductase (MCR) (Supplementary Data 10). We also search the metagenomic dataset extensively for potential unbinned mcr genes and were not able to identify any. To be certain that Brockarchaeota are not capable of producing methane we compared their gene contents to other archaea capable of methanogenesis. We found just a few potential proteins involved in methanogenesis. JZ-2_136 appears to code for three methanogenesis marker genes (Soluble P-type ATPase (arCOG01579), arCOG04853, and DUF2119 (arCOG04894), however these are also present in some other non-methanogenic archaea[10]. (Supplementary Data 10) GD2_1_47_42 is the only MAG that encodes trahydromethanopterin S-methyltransferase subunit A (MtrA). Genomes from Yunnan hot springs (DRTY-1_18, DRTY7 _35_44 and DRTY7_37) encode m17_Zn-ribbon protein that is also present in Verstraetearchaeota, Bathyarchaeota, and known methanogenic genomes.

Some Brockarchaeota code key enzymes of the tetrahydromethanopterin (H_4 MPT) methyl pathway including formylmethanofuran dehydrogenase (MRF-dehydrogenase) and the F_{420}-dependent methylene-H_4MPT dehydrogenase (MTD) (Supplementary Figure 3). MRF-dehydrogenase performs the first step in the Wolf cycle to reduce CO_2 to formylmethano-furan (MFR-CHO)[11]. In methanogenic archaea, the formyl group of the MFR-CHO is then transferred

to H_4MPT to yield CH_3-H_4MPT which ultimately is transferred to coenzyme M (HS-CoM) by the methyl-H_4MPT: coenzyme M methyltransferase (MtrA-H) complex[12] to generate a proton translocation gradient that drives ATP synthesis. According to the core-methanogenesis analysis (Supplementary Data 10), all Brockarchaeota MAGs lack the main Mtr subunits. (only GD2_1 MAG encodes MtrA), indicating that there is no coupling site for methyl-coenzyme M formation associated to an electrochemical gradient concomitant to ATP formation. Although H_4MPT methyl branch of the WLP could potentially be a natural pathway for degrading methylated compounds in Brockarchaeota, as it has been seen in other archaea (i.e. Verstraetearchaeota), and appears to be an important module for methylotrophy, our results suggest that the H_4MPT- WLP is not likely to be involved in the usage of methylated compounds in Brockarchaeota. In summary: i) there is a lack of common core marker genes specific to methanogenesis including methyl-coenzyme M reductase, ii) the lack of key ezymes of the H_4MPT involved in the transfer and reduction of C1 moieties (Ftr, Mch and Mer) iii) they lack CO-dehydrogenase−acetyl-CoA-synthase complex that leads to biomass production via carbon fixation, therefore methylotrophy cannot be coupled to energy conservation or biomass production in Brockarchaeota (Supplementary Figure 3).

Brockarchaeota encodes the two main subunits of the methyltansferase, the key enzyme of the methyltransferase system MTS: The methanol-cobalamin methyltransferase (MtaB) (Figure 3 panel A) and trimethylamine-corrinoid protein methyltransferase (MttB) (Figure 3 panel B), that are evolutionarily distinct from all known methyltransferases. The second subunit a B12-binding corrinoid protein, that once methylated, can act as a substrate for the second enzyme of the MTS that is lacking in Brockarchaeota genomes. In the case of methanol usage, this enzyme, transfers the methyl group from MtaC to coenzyme M in methanogenic archaea or tetrahydrofolate in acetogens. Based on the metabolic analyses, we hypothesized that an undescribed protein may be involved in the transfer of methylated compound from the corrinoid protein to tetrahydrofolate and follow the same H_4F assimilation route described for formate assimilation. In this way, Brockarchaeota could potentially contain convergent mechanisms for the use of formaldehyde, methanol and TMA leading to carbon assimilation, biomass production and energy conservation via acetate production.

Degradation of complex carbohydrates

Brockarchaeota genomes contain a wide variety of genes involved in the break down glycans (CAZymes; Carbohydrate-Active Enzymes) which are divided in the following classes: Carbohydrate-binding modules (CBM), Glycoside Hydrolases (GH) Glycoside Transferases (GT) and Polysaccharide Lyases (PL). A total of 301 CAZYmes-related genes were identified among the 15 Brockarchaeota genomes (Supplementary Data 12). The hot spring ones are the most metabolically versatile, encoding from 8-58 CAZYmes per genome, while GB genomes encode 4-10 (B48_G17, B27_G9 respectively). The genome DRTY.6.80 exhibits 58 CAZymes-related genes, that is more than the average of Brockarchaeota CAZymes-related genes (around 27 genes per genome).

The most diverse enzymatic class among present in Brockarchaeota genomes is GH. This group of CAZYmes is common in heterotrophs and they catalyze the hydrolysis of glycosidic bonds in primary metabolism, allowing the use of complex carbohydrates as energy sources[13]. The predicted extracellular CAZYme belong to the GH class, and they indicate that Brockarchaeota could have a key role in biomass degradation.

Five different types of GTs were detected among Brockarchaeota genomes. Interestingly, type GT4 and GT2 are the families with the highest number of enzymes in hyperthermophilic Archaea were also present in high numbers in Brockarchaeota. GT4 accounts for a total of 29 genes in the 15 MAGs, some like DRTY.6.80 encode up to 5 copies, while GT2 was present in both GB genomes and 4 hot spring MAGs. These families include activities involved in cellulose, chitin, and sucrose biosynthesis, but also in N-glycosylation Supplementary Data 9.

The genome size of known complex-carbon degrading microorganisms isolated from similar environments ranges from 2-4Mb. For example *Caldicellulosiruptor kronotskyensis*[14] isolated from hot springs (2.8M), the anaerobic, xylanolytic, extremely thermophilic bacterium, *Caldicoprobacter oshimai* (2.7 Mb) and the xylan-degrading bacterium *Petroclostridium xylanilyticum* (3.8Mb) (Yokohama et al., and Zhang et al., 2018). The genus *Caldicellulosiruptor* is globally distributed, being isolated from terrestrial geothermal hot springs in Russia, Iceland, Yellowstone National Park and New Zealand and from solar-heated mud flats in Owens Lake, CA. With optimal growth temperatures ranging from 70 to 78°C[15,16]. All members of the *Caldicellulosiruptor* genus have similar genome size ranging from 2.4 to 2.97 Mb. Similarly, Brockarchaeota genome size ranges from 0.94-2.9 Mb.

A comparison of CAZymes among members of the TACK superphylum revealed xylan degradation is unique to Brockarchaeota (Supplementary Data 12). The 3 unique CBM: (CBM67, CBM4 and CBM9), are associated with xylanases. Among the 13 unique GH (GH42, GH163, GH33, GH122, GH148, GH39, GH94, GH141, GH97, GH10+CBM22 GH149), two of them are predicted to be extracellular (GH10+CBM22, GH5+GH12) and with xylanase activity in Brockarchaeota. The carbohydrate esterase CE7 unique in Brockarchaeota catalyzes the hydrolysis of acetyl groups from polymeric xylan[17].

Degradation of detrital proteins

A total of 403 different peptidase-like genes were identified in Brockarchaeota MAGs (303 belong to known peptidase families and 98 uncharacterized). Interestingly, the most abundant peptidases in Brockachaeota lack identifiable substrates and are homologues to sequences belonging to the subfamilies S1C (*Nitrosoarchaeum limnia*), C44 (*Ignisphaera aggregans*), M50 non-peptidase homologues (*Methanothermus fervidus*), and U32 (*Aquifex aeolicus*). The number of peptidases in within individual genomes ranges from 29-98, the hot spring genotypes (DRTY-6.80, GD2_1_47_42, JZ-2. 136, QC4 _48_22 and QZM_A2) code for the most (all over 90 each). From the total number of predicted peptidases, only 11 are potentially extracellular (M28F, T03, S08A and unclassified families). Almost all Brockarchaeota MAGs, encode potential extracellular peptidases with exception of B48_G17, DRTY-6.200, DRTY7_35_44, JZ-1.89, JZZ.4 and QC4_43) (Supplementary data 13).

Catabolism of pentoses: xylose degradation.

Interestingly, Brockarchaeota not only possesses the metabolic capability to degrade hexoses via EMP pathway, however, they encode key enzymes involved in the degradation of pentoses specifically xylose. Degradation of xylose in Brockarchaeota can be possible by the presence of gene coding xylose isomerase XylA [EC:5.3.1.5] and xylulose kinase XylB [EC 2.7.1.17] (Supplementary Figure 7). These enzymes are only found in bacterial thermophiles and halophilic

archaea that can grow by fermentation of complex compounds and can degrade xylose suggesting a similar behavior in Brockarchaeota genomes (Johnsen & Schönheit 2004)

Fermentation

Reconstruction of Brockarchaeota metabolisms strongly support that this group is composed of hyperthermophile facultative and obligate anaerobic fermentative organism predicted to produce ATP by substrate level phosphorylation, producing acetate carbon dioxide and hydrogen as byproducts. Brockarchaeota are likely to produce H_2 during acetogenic fermentation given the presence of the bidirectional group 3b and 3c [NiFe]-hydrogenases (Supplementary Figure 10).

Brockarchaeota contain genes that encode enzymes for grow on organic substrates, which could be fermented to acetate (Supplementary Figure 6). The ATP conserving step of sugar or pyruvate fermentation to acetate is catalyzed by the enzyme acetate-CoA ligase (ADP-forming) present in the hot spring genomes. Acetate can also be converted back to acetyl-CoA by an acetyl-CoA synthetase (ACS) [EC:6.2.1.1] (present in GD2_147_42, JZ-1.89, DRTY-6.80, JZ-1.89, QC443_77, QZM_A248_33) suggesting that in the absence of other substrates, acetate can serve as a source of carbon and energy in hot springs Brockarchaeota genomes. Due to the acetogenic capability, we compared Brockarchaeota to other homoacetogenic microorganisms that can couple H_2-dependent CO_2 reduction with H_2-producing fermentation. Homoacetogenic archaea such as Lokiarchaeota couple the Wood–Ljungdahl pathway (WLP) with a variety of organic electron donors (for example, sugars, ethanol and lactate) (See Orsi et al., 2020). However, Brockarchaeota lacks the enzymes for methanogenesis including the methyl and carbonyl branches (Supplementary Figure 3), eliminating any possible mechanism for coupling H_2 producing fermentation with WLP. Brockarchaeota encode the four subunits of the pyruvate ferredoxin oxidoreductase (PFO), which could allow them to couple the (WL) pathway with the reductive tricarboxylic acid (rTCA). They lack key marker genes including ATP-citrate-lyase (aclAB), and carbon monoxide dehydrogenase/acetyl-CoA synthase (CODH/ACS) indicates that Brockarchaeota cannot support autotrophic metabolism.

Pyruvate ferredoxin oxidoreductase (PFO) is found in strict anaerobes and organisms that experience anoxia (including algae). PFO acts by coupling pyruvate oxidation to H_2 production ultimately generating acetyl-CoA. PFO are classified into four groups based on their subunit compositions. Consistent with the metabolic repertoire, Brockarchaeota encodes the third type of PFO, that encodes four subunits. This type of PFOs has been found in hyperthermophile anaerobes, including the fermentative archaea *Pyrococcus furiosus* and *Thermococcus litoralis*, the sulfate-reducing archaeon *Archaeoglobus fulgidus*, and the fermentative bacterium *Thermotoga maritima*[18].

Brockarchaeota genomes encode several proteins that possess the nitronate monooxygenase domain (PF03060). As it was suggested recently in Spang et al., 2019 this family may represent a novel NADH: quinone reductases, that could function in the re-oxidation of NAD(P)H generated during growth of Brockarchaeota on organic substrates as has been suggested for some fermentative members of the Asgard phylum.

Brockarchaeota from hot springs can generate Na^+ gradient using different proton pumps allowing for Na^+-driven synthesis of ATP via a V-type ATPase. For example, they encode the natA sodium transport system ATP-binding protein [EC:7.2.2.4] (Figure 3), that is active in the presence of ethanol[19] and the K(+)-stimulated pyrophosphate-energized sodium pump encode membrane-bound proton-translocating inorganic pyrophosphatases (HppA), which might have a crucial role in anaerobic metabolism[20] and may allow energy conservation from hydrolysis of pyrophosphate in Brockarchaeota couple with the electron bifurcation complex. The extent to

which this mechanism is associated with energy conservation mechanism is still unclear. Brockarchaeota genomes appear to encode a wide repertoire of ATPases: i) the plasma-membrane proton-efflux P-type ATPase [EC:7.1.2.1] (K01535) (only present in the hot spring genomes), ii) Zn2+/Cd^{2+}-exporting ATPase [EC:7.2.2.12 7.2.2.21] (K01534) only present in DRTY7.37 and finally the V/A-type H+/Na+-transporting ATPase (present in GB and hot spring genomes). The existence of ATPase in Brockarchaeota suggests that members of this latter group have the additional ability to couple acetogenic fermentation to membrane potential generation of a transmembrane ion gradient across the membrane.

The enzymatic repertoire differs between MAGs from GB and hot springs suggesting they have different anaerobic physiological lifestyles; this could also be the reflection of low number of genomes from deep-sea sediments. The recovery of additional genomes will resolve this in the future. GB genomes appear to be obligately fermenting organisms that rely mostly on substrate level phosphorylation since they lack all the complexes for the respiratory chain with exception of the ATPase. In contrast, hot spring genomes appear to have mechanisms to increase their ATP yield including the use of geothermally derived inorganic substrates as possible terminal electron acceptors.

Butanol degradation

Brockarchaeota genomes from hot springs encoded a butanol dehydrogenase BDH (Supplementary Figure 4) that catalyzes the reversible conversion of butyraldehyde to butanol, oxidizing NAD(P)H. Brockarchaeota BDHs are homologues to obligately anaerobic, thermophilic bacteria that can degrade complex of complex plant saccharides such as xylan (i.e *Caldicoprobacter oshimai Hungateiclostridium thermocellum* or cellulose (*Hungateiclostridium alkalicellulosi*) (First, we looked for the genes described in the obligately anaerobic bacterium *Clostridium acetobutylicum*[,34] since it is one of the few organisms known to produce butanol as a major fermentation product. We also searched the pathway involved in butanol and isopropanol production described in *Saccharomyces cerevisiae* (Branduardi et al., 2013). We found that Brockarchaeota genomes lacks the key enzymes involved in the fermentation of pyruvate to butanol (butanal dehydrogenase, butyryl-coA dehydrogenase, enoyl-CoA dehydratase, 3-hydroxyacyl-CoA dehydrogenase). However, most of the genomes encode a putative aldehyde dehydrogenase (PF00171) that could convert butyraldehyde to butyric acid. Also, we found a putative enoyl-CoA hydratase/isomerase protein that is only encoded in bin JZ-1.89, that could be involved in further processing of the butyric acid molecule. Our results suggest an alternative pathway for butanol oxidation that still remains unresolved (Supplementary Figure 5).

Detoxification or energy conservation mechanisms

Arsenate.

The genetic system for arsenic resistance is present in Brockarchaeota genomes. This detoxification system acts by decreasing the intracellular arsenic concentration by pumping out arsenate that enters the cell, thus preventing the metals from accumulating and denaturing proteins. (See Nunes et al., 2014). The intracellular dependent arsenate reductase (ArsC, K03741) that catalyzes the reduction of arsenate AsO_4^{3-} to arsenite $As(OH)_3$ (Figure 4), is present in most hot spring genomes (Supplementary Data 6). Phylogeny of Brockarchaeota ArsC (Supplementary Figure 9) indicates that they belong to a deep uncharacterized branch of Thioredoxin-coupled clade, that has been mainly described in Firmicutes (Silver et al., 2005). In agreement with the

geothermal origin of Brockarchaeota genomes, homologous ArsC sequences recovered from geothermally active environments belonging to uncultured Bathyarchaeota or Thaumarchaeota, which could potentially be Brockarchaeota, or have a similar arsenate metabolism (See Colman et al., 2019 and Zhou et al., 2020).

In biochemical and molecular terms, arsenate AsO_4^{3-} which is similar to phosphate, can enter the cell by phosphate transporter present in Brockarchaeota genomes (Supporting Data 5). Once in the cytoplasm, the conversion of arsenate AsO_4^{3-} to arsenite $As(OH)_3$ by thioredoxin arsenate reductase, the non-toxic arsenite is then exported from the cell by the arsenite efflux transporter (asrA, K01551 and asrB K03893). The presence of this energy-dependent efflux process and related detoxification enzymes (see Supplementary Data 6), in hot springs genotypes could be associated to energy conservation mechanism. For example, some anaerobic bacteria like *Desulfovibrio* strain Ben-RA can reduce arsenate in the presence of *asrC*-like genes (Rabus et al., 2015). Subsequently, molecular genetics and biochemical studies with *D. alaskensis* G20 demonstrated that ArsC functions as an efficient arsenate reductase with electrons delivered from thioredoxin, however the exact molecular mechanism of this process is unknown (Nunes et al., 2014)

Mercury.

Three hot springs Brockarchaeota genomes (DRTY735_44, DRTY-1.18 and DRTY7.37) encode mercuric reductase (MerA), the central enzyme in the microbial mercury detoxification system. MerA transforms the extremely toxic Hg (II) to metallic Hg(0), a possible mechanism for in mercury detoxification. MerA phylogeny indicates that Brockarchaeota possess a previously uncharacterized class of MerA, which are related to other archaea (Supplementary Figure 8).

Sulfur cycling

Thiosulfate is a significant intermediate in the sulfur cycle of anoxic marine and freshwater sediments, where it is involved in reduction, oxidation, and disproportionation pathways[23]. JZ-2.136 is the only bin that encodes Rhodanese (PF00581) implicated in the disproportionation of thiosulfate for energy conservation purposes[24]. Biochemical characterization of *Aquifex aeolicus*, isolated from an hydrothermal system indicate that this bacterium can use either tetrathionate ($S_4O_6^{2-}$), polysulfide or thiosulfate ($S_2O_3^{2-}$) as electron donors using the rhodanese pathway, however despite numerous studies, the physiological role of this pathway remain unclear and are still widely debated[24], and therefore the role of Rhodanase in Brockarchaeota remains unclear. Similar to other heterotrophic fermentative hyperthermophilic archaea, Brockarchaeota might be able to reduce elemental sulfur during fermentative growth and produce H_2S due to the presence of [NiFe] Group 3b hydrogenases (Supplementary Figure 10). During carbohydrate fermentation in the absence of sulfur, [NiFe] Group 3b hydrogenase can catalyze the production of H_2 with NADPH or NAD(P)H as the electron donor. However, in the presence of sulfur, Brockarchaeota from might have the ability to reduce sulfur using H_2 or organic substrates as electron donors, a widespread physiology in hyperthermophilic archaea living in geothermally active environments (volcanic habitats, hots springs or marine sediments). Another indicator of sulfur usage as a source of energy was by scoring Brockarchaeota genomes with MEBS (See methods and Supplementary Data 6). QZM_A2 and QZM_A3 genomes display a comparable sulfur score (6.459 and 6.591 respectively: to some known elemental sulfur reducers from the order Thermococcales and Thermoanaerobacterales.

Hydrogen utilization

Hydrogen is found at significant levels in most hydrothermal systems due to volcanic outgassing or abiotic production. Since [NiFe] Group 3b hydrogenase can also catalyze the reverse reaction of H_2 oxidation using NADP+ or NAD(P)+ as an electron acceptor, it is possible that Brockarchaeota might be the use of hydrogen gas as electron donor. Brockarchaeota from hot springs might switch metabolism depending on the availability of external electron acceptors, is the presence of the oxygen-tolerant group 3d [NiFe]-hydrogenases (Supplementary Figure 10). This group is suggested to interconvert electrons between NAD(P)H and H_2 depending on the availability of electron acceptors. Consistently, 3d [NiFe]-hydrogenases are found to be abundant at metagenomic level in hot springs where it suggested that this could be the reason why microbial communities in these geothermally active systems are relatively stable despite pO_2 fluctuations (See Greening, et al., 2016)

Supplementary Figures

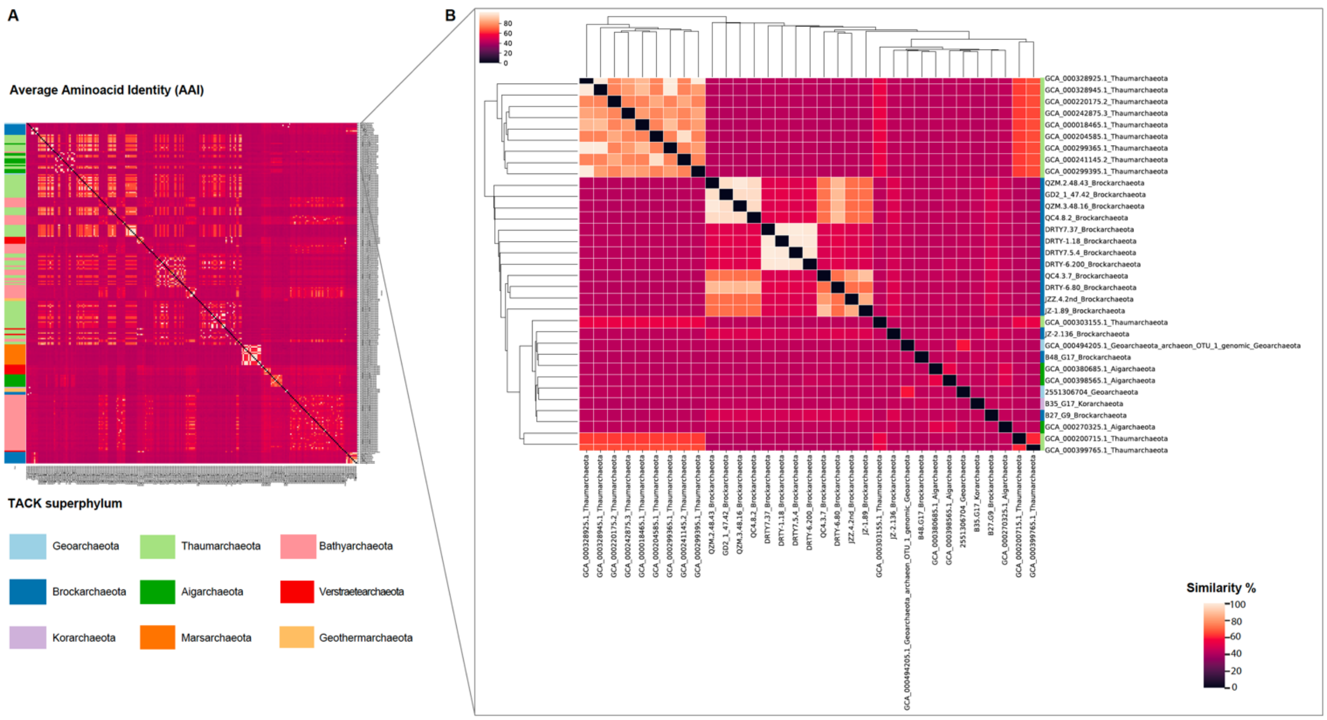

Supplementary Figure 1. Average amino acid identity (AAI) comparison of Brockarchaeota genomes and phylogenetically related TACK phyla A) Hierarchical clustering heatmap based on average amino acids identity for each genome pair of genomes described in Supplementary Data 4. Genome self-comparisons are shown in the black square diagonal. Analysis was done using compare v0.0.23 (https://github.com/dparks1134/CompareM) B) Hierarchical clustering heatmap based on average amino acids identity for each genome pair of closely related genomes o Brockarchaeota based on panel A. Selected genomes include Geoarchaeota, Aigarchaeota and Thaumarchaeota phyla. The color code indicates the AAI (%) from 0-100 (red to white respectively).

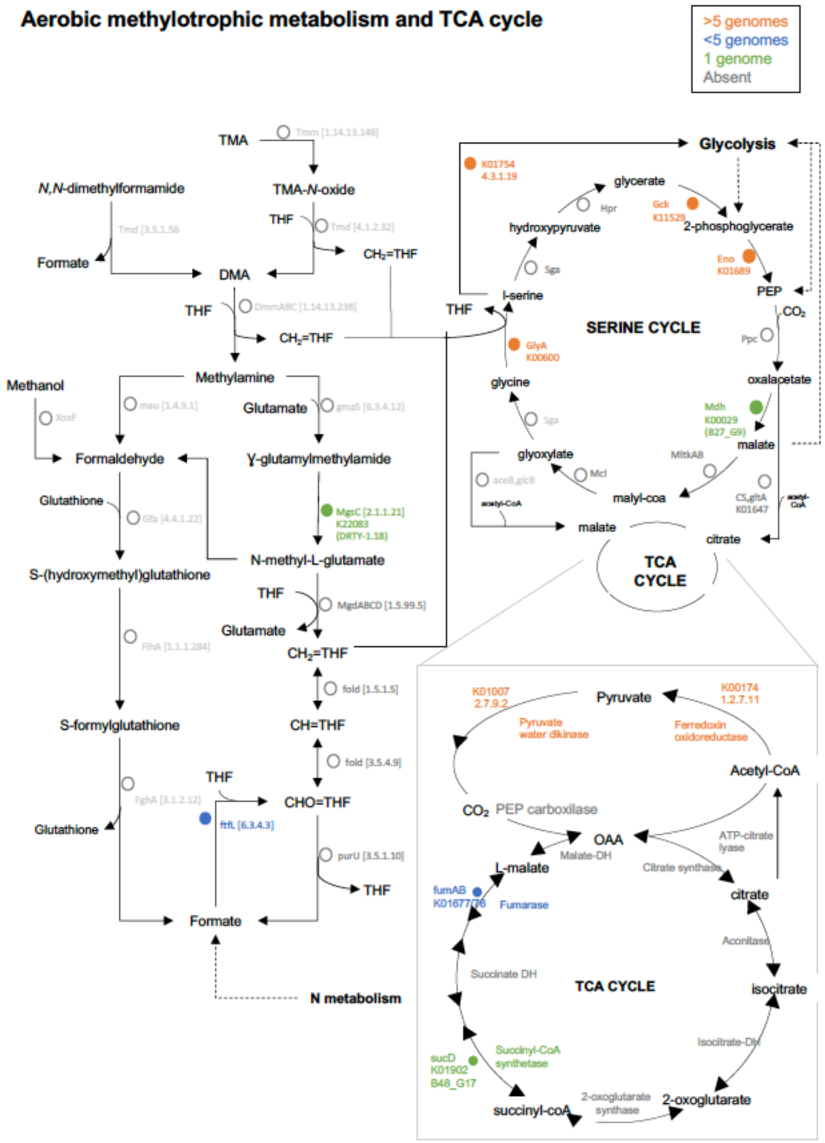

Supplementary Figure 2. Enzymatic steps involved in aerobic methylotrophy described in Dziewit et al 2015[25]. In the serine cycle, after formate condensation, methylene-H4F transfers its C1 moiety to glycine to generate serine. Serine is then converted to phosphoenolpyruvate (PEP), carboxylated to oxaloacetate, and subsequently reduced to malate. Malate is then activated by coenzyme A and cleaved to glyoxylate, which regenerates glycine and acetyl-CoA, which serves as a biomass precursor. Malate dehydrogenase (oxaloacetate-decarboxylating) (NADP+) [EC:1.1.1.40] K00029 is only present only in one B27_G9 genome (Supplementary Data 6).

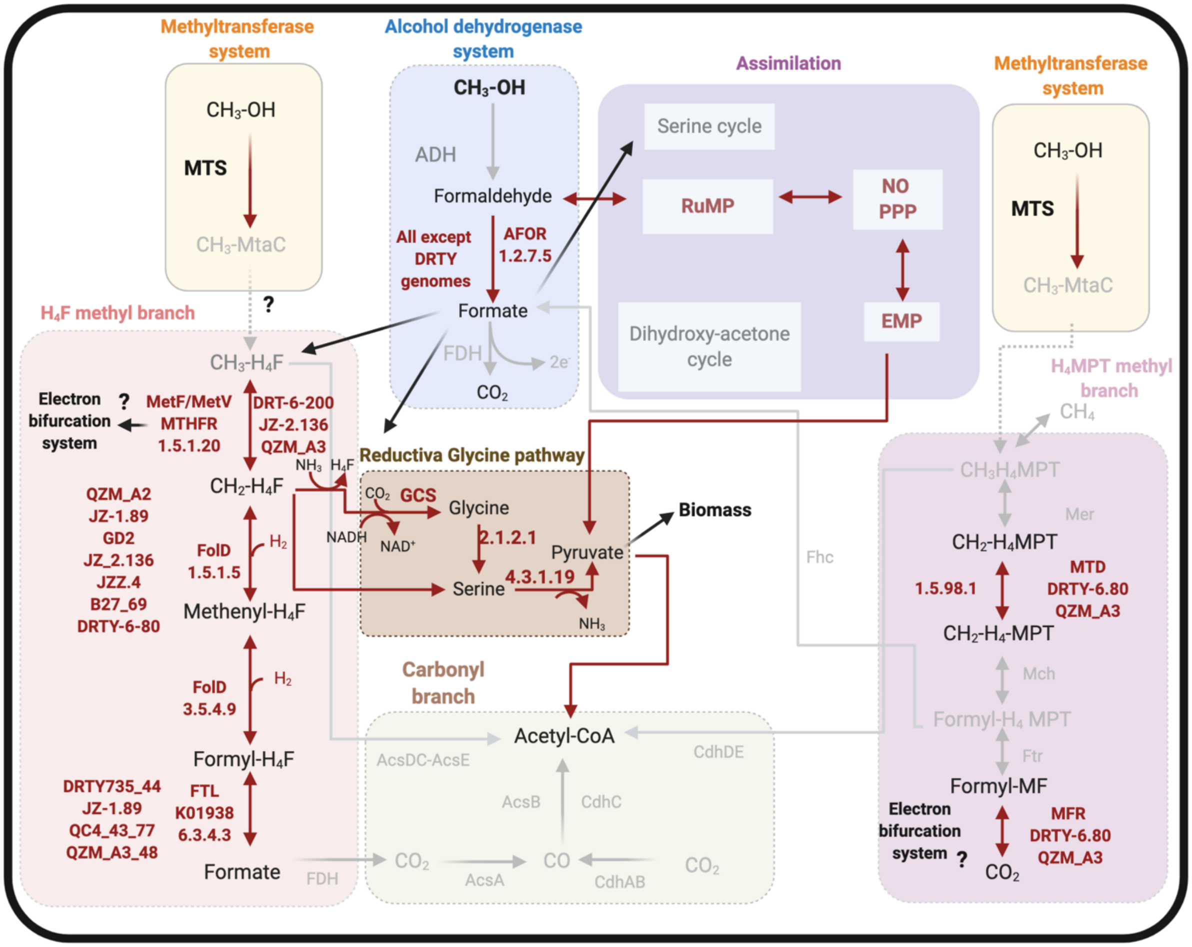

Supplementary Figure 3. Predicted pathways for the usage of methylated compounds in several phylogenetically unrelated organisms (Bacteria and archaea including Brockarchaeota). Alcohol dehydrogenase system hypothesized for the methanol-degrading sulfate reducer *Desulfotomaculum kuznetsovii* (See Sousa et al. 2018) (Blue panel). Similar to *D. kuznetsovii*, Brockarchaeota genomes possess an alcohol dehydrogenase (ADH) however, according to the ADH phylogeny (Supplementary figure 4), Brockarchaeota ADH's are more evolutionary related to butanol-type alcohol dehydrogenase (see Supporting Figure 5, for the proposed butanol metabolism). Black arrows indicate possible formate assimilation pathways described in the text. Abbreviations: Glycolysis via Embden–Meyerhof–Parnas (EMP Supplementary Figure 6) , Non-Oxidative Pentoses Phosphate Pathway (NOPPP), and Ribulose monophosphate Pathway (RuMP) (Supplementary Figure 7); Methyltransferase system (MTS), Formate dehydrogenase (FDH), tungsten-dependent aldehyde ferredoxin oxidoreductase (AFOR);H_4MPT methyl branch (tetrahydromethanopterin (H_4MPT) , H_4F methyl branch (tetrahydrofolate (H_4F); Glycine cleavage system (GCS), methylene-H_4F reductase (NADPH) (MetFV); methylenetetrahydrofolate dehydrogenase (NADP+)/methenyltetrahydrofolate cyclohydrolase FolD, Formate-tetrahydrofolate ligase (FTL), F_{420}-dependent methylene-H_4MPT dehydrogenase (MTD), formylmethanofuran dehydrogenase (MRF). Created with BioRender.com

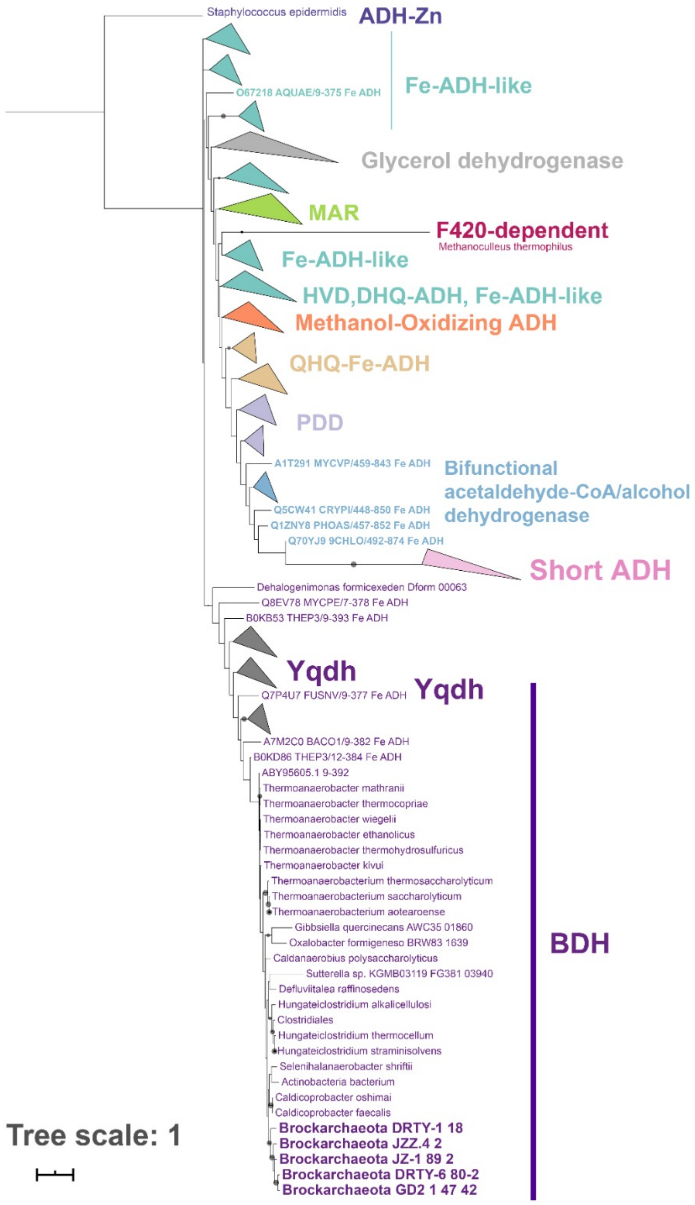

Supplementary Figure 4. Phylogeny and classification of alcohol dehydrogenases (ADH) amino acid sequences found in Brockarchaeota genomes. The sequences were obtained from a BLASTp analysis using ADHs from Brockarchaeota as query sequences. Reference ADHs were obtained as follows: short chain ADH (PF00106), Fe-containing ADH (PF00465) methanol dehydrogenase. was obtained from *Desulfotomaculum kuznetsovii* according to Sousa et al., 2018. Sequences described in Radianingtyas et al., 2003 [26] were obtained from UniProtKB and KEGG databases. A total of 301 sequences, including 5 from Brockarchaeota MAGs were aligned using MAFFT v7.450 (default parameters). The alignment was masked (50% Gaps) in Geneious Prime 2020.0.5 and manually refined. The phylogenetic tree was generated using a maximum likelihood-based approach using RAxML v8.2.10, called as: raxmlHPC-PTHREADS-AVX -f a -m PROTGAMMAAUTO -N autoMRE -p 12345 -x 12345 -s. ADH fasta sequences and phylogenetic tree are available from the corresponding author on request.

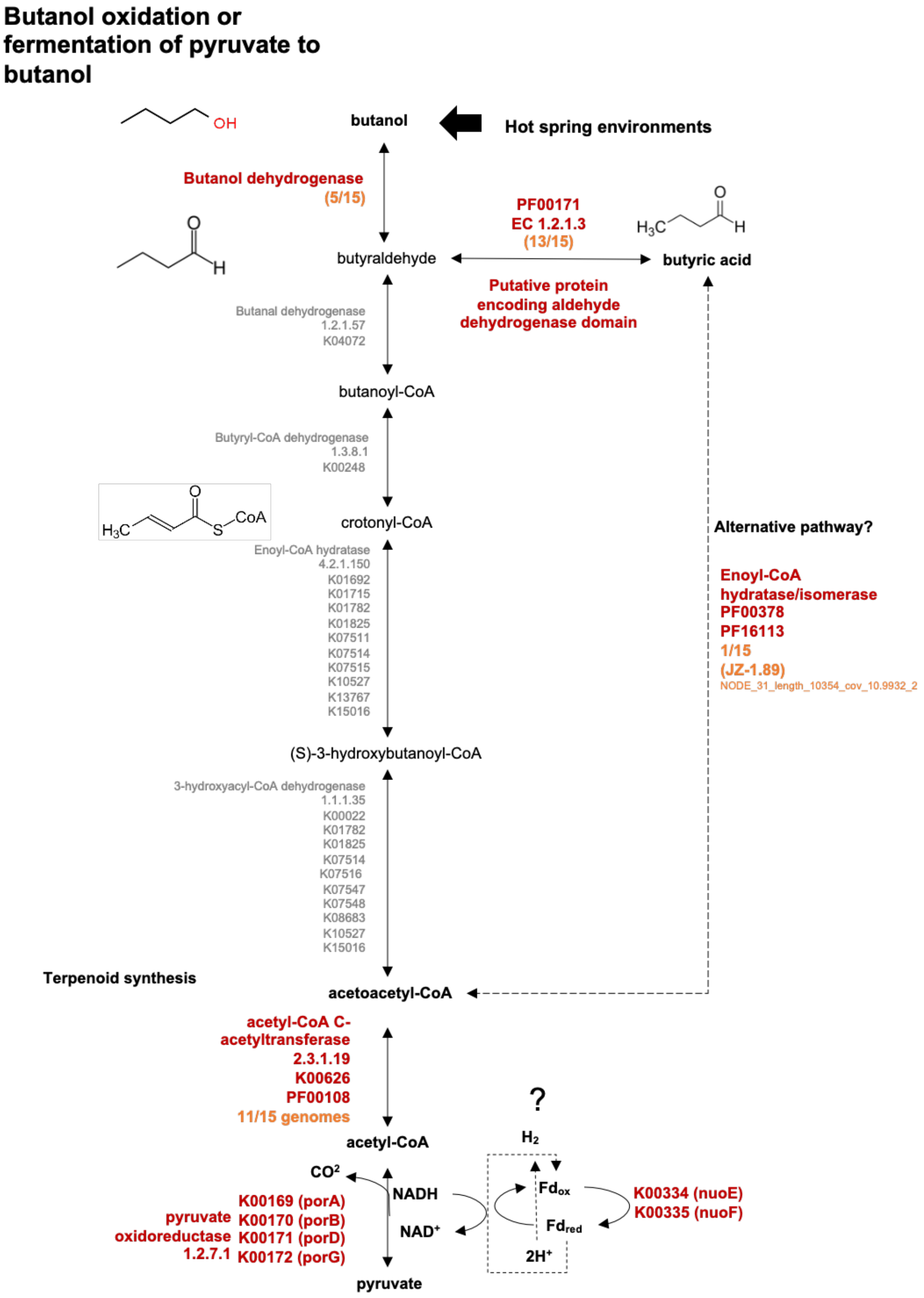

Supplementary Figure 5. Proposed pathway for butanol oxidation in Brockarchaeota genomes from hot springs where butanol dehydrogenase (BDH) was present. Brockarchaeota lack the set of enzymes involved in the fermentation of pyruvate to butanol, yet they encode a putative aldehyde dehydrogenase (PF00171) that could convert butyraldehyde to butyric acid. Red colors indicate the present enzymes in Brockarchaeota genomes. Orange colors indicate the number of Brockarchaeota genomes encoding that enzymatic step. The steps involved in further conversions of butyric acid still remain unresolved.

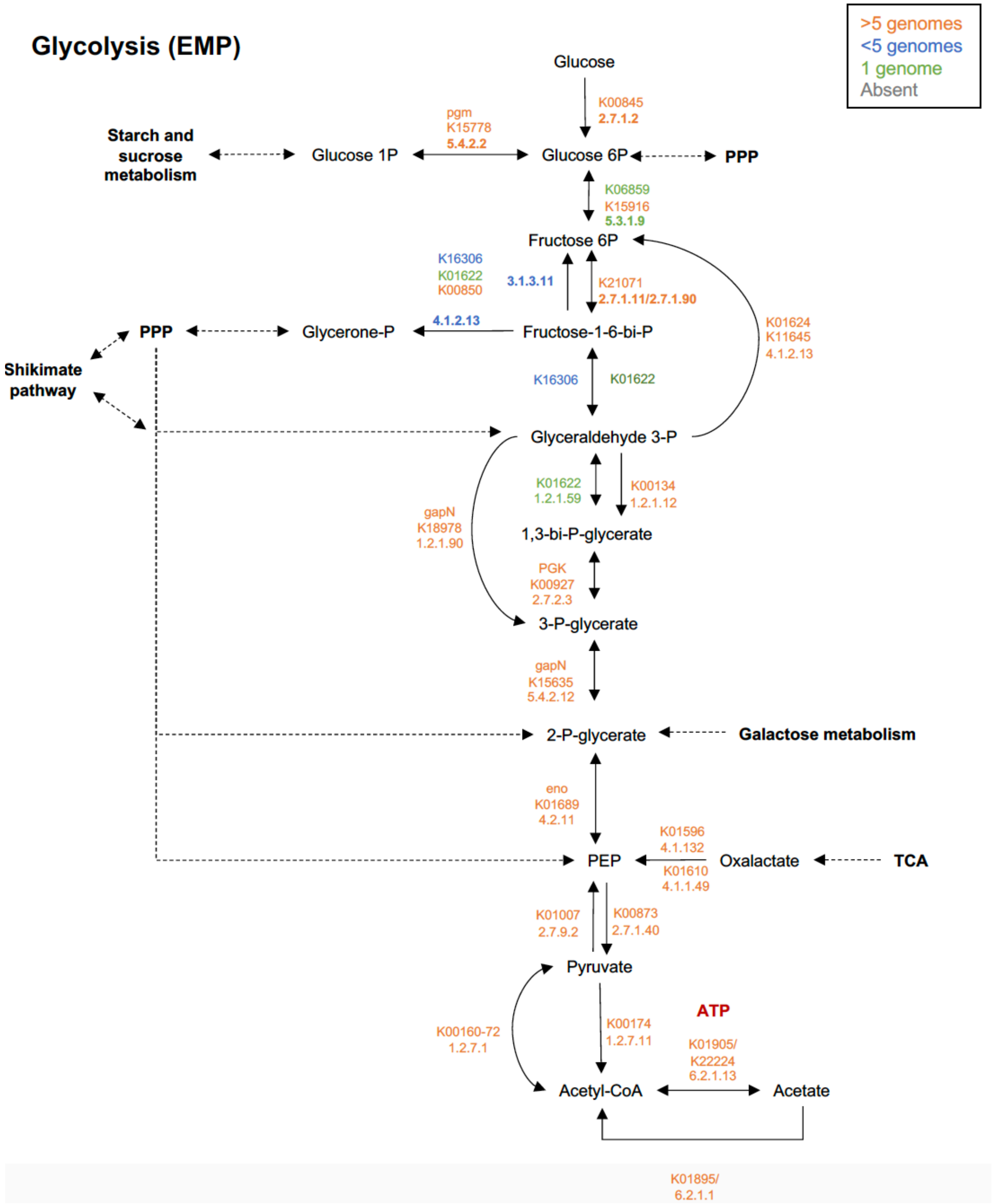

Supplementary Figure 6. Glycolytic Embden-Meyerhof pathway (EMP) found in Brockarchaeota genomes. Phosphomannomutase/Phosphoglucomutase (pgm) [EC:5.4.2.2] is a branch point between central metabolic pathways and the polysaccharide synthesis pathways. Enzymes detected in only one MAG: glucose-6-phosphate isomerase, archaeal (K06859) [EC:5.3.1.9] present in JZ-2-136; Fructose 1,6-bisphosphate aldolase/phosphatase (K01622) [EC:4.1.2.13 3.1.3.11] present in B48_G17; Glyceraldehyde-3-phosphate dehydrogenase (NADP+) [EC:1.2.1.9] (K00131), present in JZZ.4. See Supplementary Data 6 for more details.

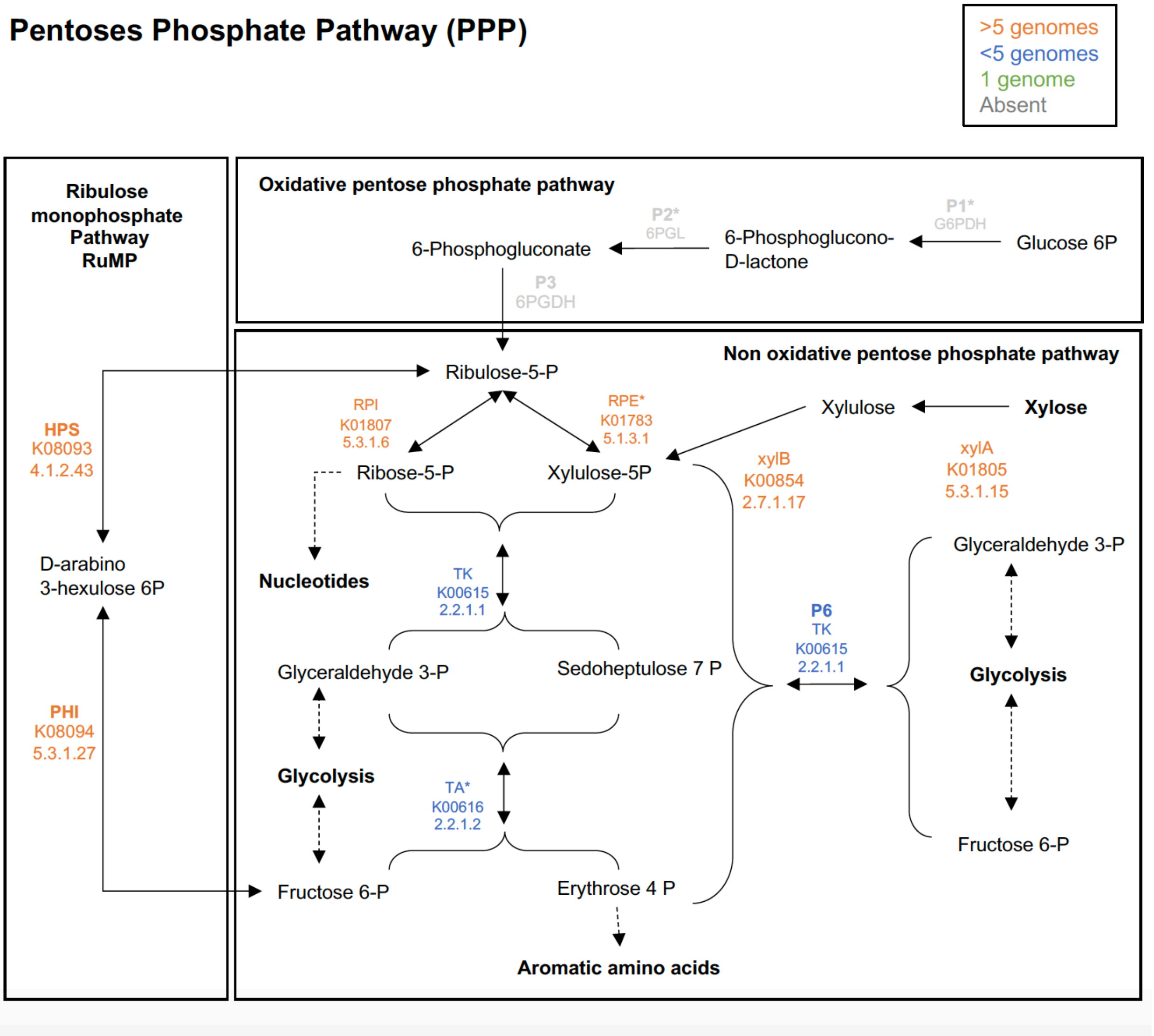

Supplementary Figure 7. Pathways for pentose formation and Ribulose monophosphate Pathway RuMP in Brockarchaeota. Asterisks indicate enzymes not identified or common in archaea according to ref.[1]. Enzymatic steps are found in Supplementary Table 6. Abbreviations: G6DPH, glucose-6-phosphate dehydrogenase; 6PGL, 6-hosphogluconate-D-lactone; 6PGDH 6-phosphogluconate dehydrogenase; RPI, ribose-5-phosphate isomerase; RPE ribose-5-phosphate-3-epimerae; TK, transketolase; TA, transaldolase; HPS,3-hexulose-6-phosphate synthase, PHI, 5-phospho-3-3hexuloisomerase

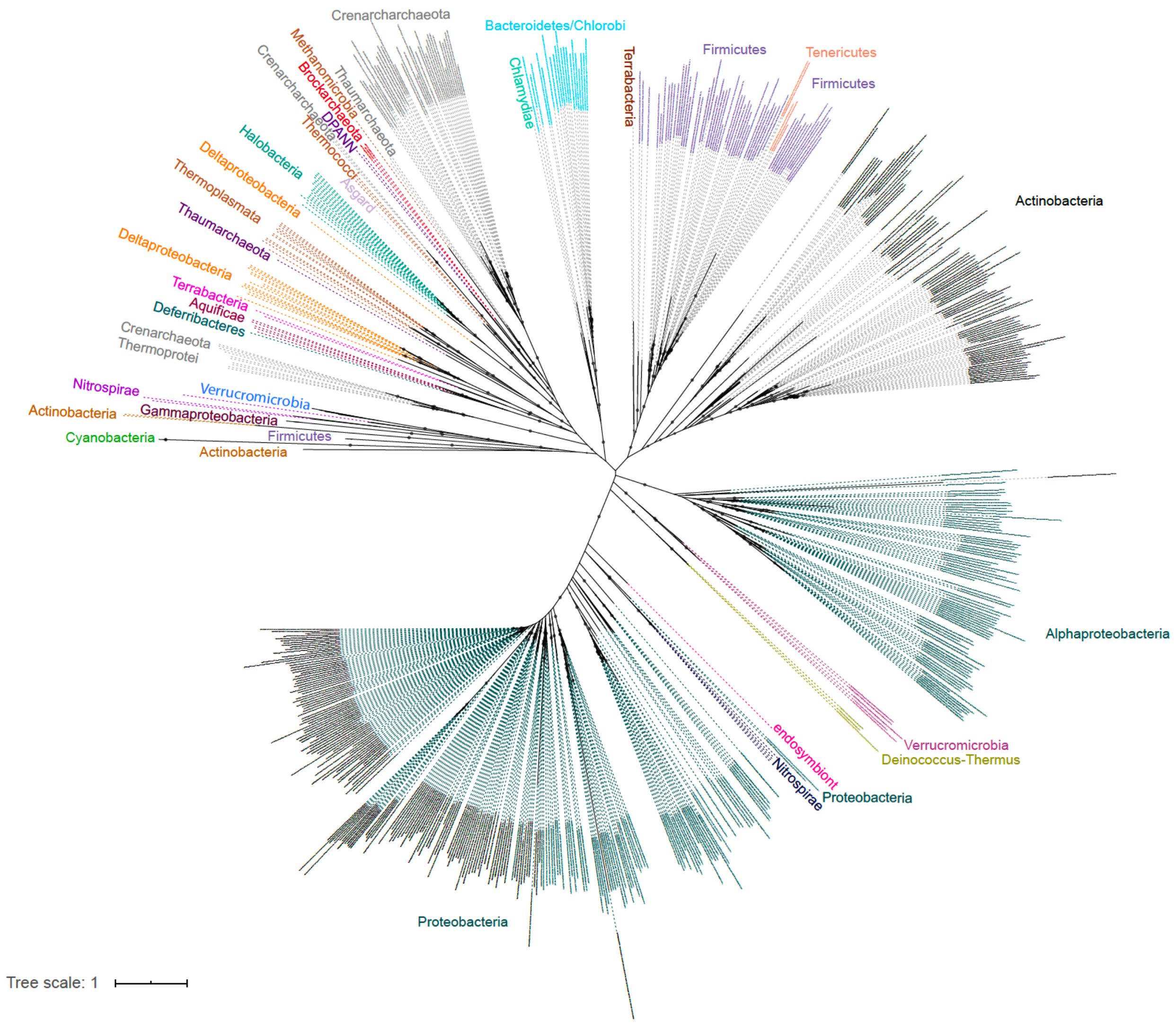

Supplementary Figure 8. Phylogenic classification of Mercury (II) reductase [EC 1.16.1.1], MerA from Brockarchaeota genomes. The maximum likelihood tree was generated from an alignment of 628 publicly available mercury reductase MerA sequences, with 3 from Brockarchaeota MAGs. Publicly available set of mercury reductases were obtained from Boyd & Barkay 2012, and the UniProtKB database (https://www.uniprot.org/). Sequences were aligned using MAFFT v7.450 (default parameters). The alignment was masked (50% Gaps) in Geneious Prime 2020.0.5 and manually refined. The phylogenetic tree was generated using a maximum likelihood-based approach using RAxML v8.2.10, called as: raxmlHPC-PTHREADS-AVX -f a -m PROTGAMMAAUTO -N autoMRE -p 12345 -x 12345 Fasta sequences and phylogenetic tree are available from the corresponding author on request. Interactive version of the tree can be found here https://itol.embl.de/tree/1872071882924494160193982‍9

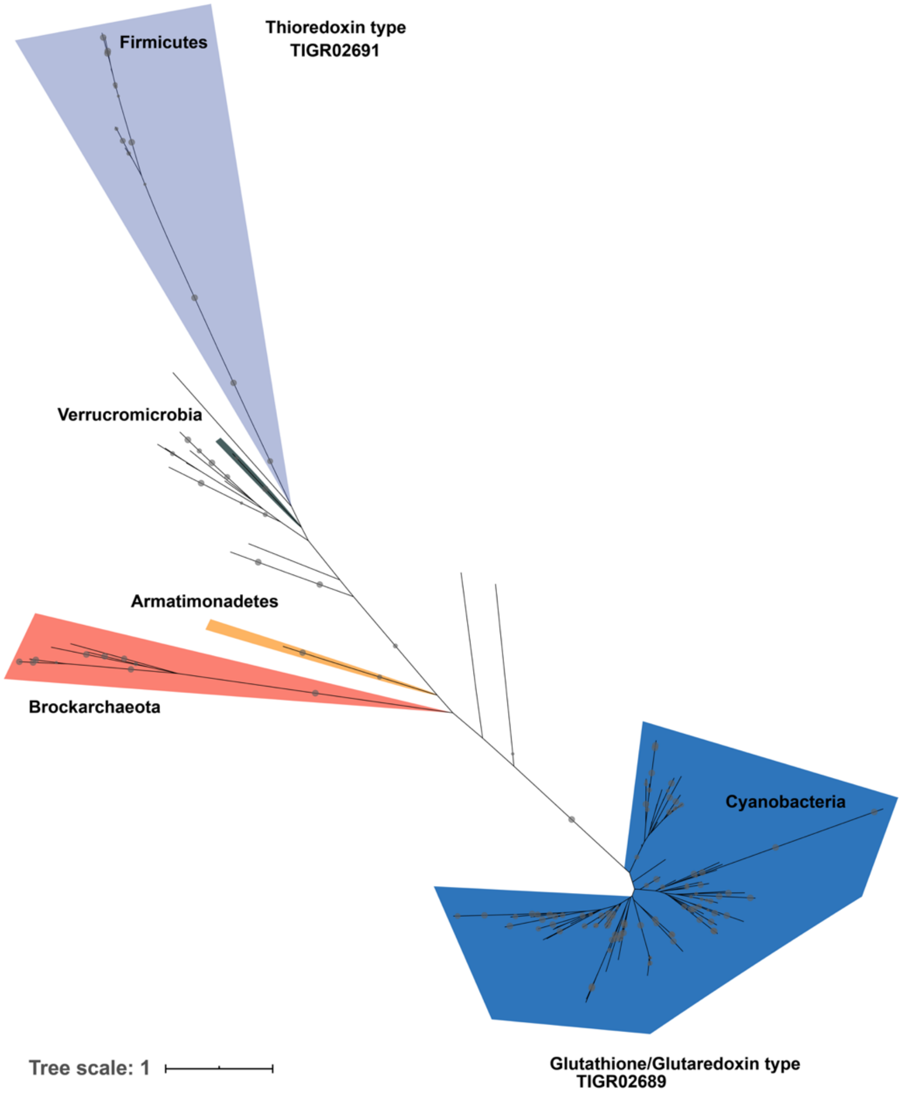

Supplementary Figure 9. Phylogenic classification of Brockarchaeota arsenate reductases (ArsC). A total of 464 sequences, including 11 from Brockarchaeota MAGs were aligned using MAFFT v7.450 (default parameters). Alignment generated with a dataset of reference arsenate reductases containing: 343 arsenate reductase, glutathione/glutaredoxin type [EC 1.20.4.1] (TIGR02689) and 39 arsenate reductase thioredoxin [EC 1.20.4.4] (TIGR02691) were obtained from Uniport (https://www.ebi.ac.uk/interpro/entry/tigrfams/TIGR02691/UniProt/#table); 61 arsenate reductase sequences were obtained from Jackson and Douglas 2003 Supplementary Table 1; 10 arsenate reductases were arsenic sequences were obtained from a BLASTp analysis using *arsC* from Brockarchaeota as query sequences. The alignment was masked (50% Gaps) in Geneious Prime 2020.0.5 and manually refined. The phylogenetic tree was generated using a maximum likelihood-based approach using RAxML v8.2.10, called as: raxmlHPC-PTHREADS-AVX -f a -m PROTGAMMAAUTO -N autoMRE -p 12345 -x 12345. Branches are color-coded according to taxonomy and enzyme type. Fasta sequences and phylogenetic tree are available from the corresponding author on request. Interactive version of the tree can be found here https://itol.embl.de/tree/7915458241171271605026109

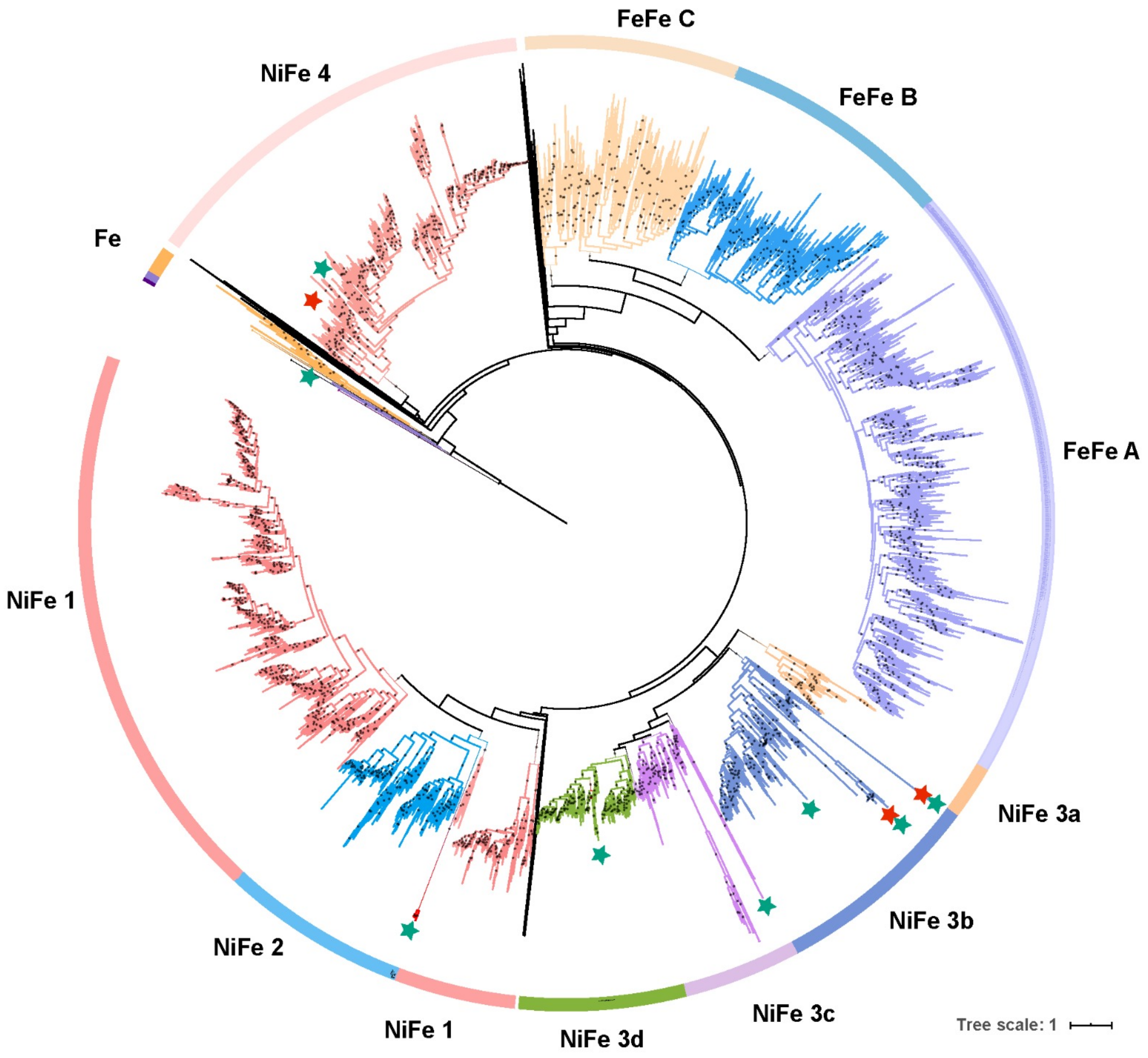

Supplementary Figure 10. Classification and phylogeny of hydrogenases found in Brockarchaeota genomes. Maximum likelihood tree aligned with a dataset of hydrogenases belonging to each class encircled and colored according to each of the IV groups of hydrogenases described in Søndergaard et al., 2016 and Greening et al., 2016. Branches with red and green stars indicate hydrogenases found in Brockarchaeota genomes from deep sea sediments and hots springs respectively. A total of 41 hydrogenases were detected in Brockarchaeota MAGs and were aligned with 3250 reference hydrogenases using MAFFT v7.450 (default parameters) and redefined with MUSCLE v3.8.425 (default parameters). The alignment was masked (50% Gaps) in Geneious Prime 2020.0.5 and manually refined. The phylogenetic tree was generated using a maximum likelihood-based approach using RAxML v8.2.10, called as: raxmlHPC-PTHREADS-AVX -f a -m PROTGAMMAAUTO -N autoMRE -p 12345 -x 12345 -s. Interactive version of the tree can be found here https://itol.embl.de/tree/97982318242371572399620

Supplementary References

1. Bräsen, C., Esser, D., Rauch, B. & Siebers, B. Carbohydrate metabolism in archaea: Current insights into unusual enzymes and pathways and their regulation. *JAMA Ophthalmol.* **132**, 326–331 (2014).
2. Say, R. F. & Fuchs, G. Fructose 1,6-bisphosphate aldolase/phosphatase may be an ancestral gluconeogenic enzyme. *Nature* **464**, 1077–1081 (2010).
3. Anderson, I. *et al.* Genomic characterization of methanomicrobiales reveals three classes of methanogens. *PLoS One* **4**, (2009).
4. Orita, I. *et al.* The archaeon Pyrococcus horikoshii possesses a bifunctional enzyme for formaldehyde fixation via the ribulose monophosphate pathway. *J. Bacteriol.* **187**, 3636–3642 (2005).
5. Moran, J. J. *et al.* Formaldehyde as a carbon and electron shuttle between autotroph and heterotroph populations in acidic hydrothermal vents of Norris Geyser Basin, Yellowstone National Park. *Extremophiles* **20**, 291–299 (2016).
6. Bar-Even, A. Formate Assimilation: The Metabolic Architecture of Natural and Synthetic Pathways. *Biochemistry* **55**, 3851–3863 (2016).
7. Mock, J., Wang, S., Huang, H., Kahnt, J. & Thauer, R. K. Evidence for a hexaheteromeric methylenetetrahydrofolate reductase in Moorella thermoacetica. *J. Bacteriol.* **196**, 3303–3314 (2014).
8. Buckel, W. & Thauer, R. K. Flavin-based electron bifurcation, ferredoxin, flavodoxin, and anaerobic respiration with protons (Ech) or NAD+(Rnf) as electron acceptors: A historical review. *Front. Microbiol.* **9**, (2018).
9. Sánchez-Andrea, I. *et al.* The reductive glycine pathway allows autotrophic growth of Desulfovibrio desulfuricans. *Nat. Commun.* **11**, 1–12 (2020).
10. Borrel, G. *et al.* Wide diversity of methane and short-chain alkane metabolisms in uncultured archaea. *Nat. Microbiol.* **4**, 603–613 (2019).
11. Rana, A. & Dey, A. Theoretical exploration of the mechanism of formylmethanofuran dehydrogenase: the first reductive step in CO2 fixation by methanogens. *J. Biol. Inorg. Chem.* **21**, 703–713 (2016).
12. Thauer, R. K., Kaster, A. K., Seedorf, H., Buckel, W. & Hedderich, R. Methanogenic archaea: Ecologically relevant differences in energy conservation. *Nat. Rev. Microbiol.* **6**, 579–591 (2008).
13. Moracci, M., Mose, B. C., Ii, F. & Universitario, C. Extremophiles Handbook. *Extrem. Handb.* (2011) doi:10.1007/978-4-431-53898-1.
14. Blumer-Schuette, S. E. *et al.* Complete genome sequences for the anaerobic, extremely thermophilic plant biomass-degrading bacteria Caldicellulosiruptor hydrothermalis, Caldicellulosiruptor kristjanssonii, Caldicellulosiruptor kronotskyensis, Caldicellulosiruptor owensensis, and Caldic. *J. Bacteriol.* **193**, 1483–1484 (2011).
15. Straub, C. T. *et al.* Quantitative fermentation of unpretreated transgenic poplar by Caldicellulosiruptor bescii. *Nat. Commun.* **10**, 1–6 (2019).
16. Yang, S. J. *et al.* Classification of 'Anaerocellum thermophilum' strain DSM 6725 as Caldicellulosiruptor bescii sp. nov. *Int. J. Syst. Evol. Microbiol.* **60**, 2011–2015 (2010).
17. Sista Kameshwar, A. K. & Qin, W. Understanding the structural and functional properties of carbohydrate esterases with a special focus on hemicellulose deacetylating acetyl xylan esterases. *Mycology* **9**, 273–295 (2018).

18. Mai, X. & Adams, M. W. W. Characterization of a fourth type of 2-keto acid-oxidizing enzyme from a hyperthermophilic archaeon: 2-Ketoglutarate ferredoxin oxidoreductase from Thermococcus litoralis. *J. Bacteriol.* **178**, 5890–5896 (1996).
19. Cheng, J., Guffanti, A. A. & Krulwich, T. A. A two-gene ABC-type transport system that extrudes Na+ in Bacillus subtilis is induced by ethanol or protonophore. *Mol. Microbiol.* **23**, 1107–1120 (1997).
20. Pereira Cardoso, I. *et al.* A comparative genomic analysis of energy metabolism in sulfate reducing bacteria and archaea. **2**, 1–22 (2011).
21. Girbal, L., Croux, C., Vasconcelos, I. & Soucaille, P. Regulation of metabolic shifts in Clostridium acetobutylicum ATCC 824. *FEMS Microbiol. Rev.* **17**, 287–297 (1995).
22. Qi, F. *et al.* Improvement of butanol production in Clostridium acetobutylicum through enhancement of NAD(P)H availability. *J. Ind. Microbiol. Biotechnol.* **45**, 993–1002 (2018).
23. Stoffels, L., Krehenbrink, M., Berks, B. C. & Unden, G. Thiosulfate reduction in salmonella enterica is driven by the proton motive force. *J. Bacteriol.* **194**, 475–485 (2012).
24. Aussignargues, C. *et al.* Rhodanese functions as sulfur supplier for key enzymes in sulfur energy metabolism. *J. Biol. Chem.* **287**, 19936–19948 (2012).
25. Dziewit, L. *et al.* Genome-guided insight into the methylotrophy of Paracoccus aminophilus JCM 7686. *Front. Microbiol.* **6**, 1–13 (2015).
26. Radianingtyas, H. & Wright, P. C. Alcohol dehydrogenases from thermophilic and hyperthermophilic archaea and bacteria. *FEMS Microbiol. Rev.* **27**, 593–616 (2003).
