# Supplementary material for: Brockarchaeota, a novel archaeal phylum with unique and versatile carbon cycling pathways
Source: Nat Commun. 2021 Apr 23;12:2404. doi: 10.1038/s41467-021-22736-6 (PMC8065059; doi:10.1038/s41467-021-22736-6)
Supplement: Supplementary file 2 — Description of Additional Supplementary Files [file 41467_2021_22736_MOESM2_ESM.pdf]

Description of Additional Supplementary Files

File Name: Supplementary Data 1
Description: *Excel File.* Sheet Data: Physicochemical characteristics of samples where the MAGs in this study were obtained. n.a: not available data. Sheet Stats: Extended genomic statistics des cribed in Table 1

File Name: Supplementary Data 2
Description: *Excel file.* Relative abundance of the microbial community samples where Brockarchaeot a MAGs where recovered. The relative abundance of the MAGs from deep sea samples was obtained from Supplementary Data 3 in Dombrowski et al., 2018. Only samples G9 and G7 are shown, and the data i s sorted according to the relative abundance of those corresponding samples. Guaymas samples and in cudes post publication manual refinement in the taxonomy according to the recent archaea tree of life Bak er et al., 2020. The relative abundance of MAGs from Tenghchong samples was computed using MetaGai a bin_abundance.py script (https://github.com/valdeanda/MetaGaia ). Briefly, for each MAG, total length of mapped reads for individual scaffolds (mapped reads using BWA algorithm) is summed up and the total is then divided by the MAG size in bp. This number is then divided by the total number of reads to obtain t he relative abundance. The final relative abundance is multiply it by 100000000 for readability purposes. F or the Tibet samples, the genome bins obtained for a given sample, the sequencing coverage was determin ed by read mapping using Bowtie2 and coverage calculation using the jgi_summarize_bam_contig_depths script from MetaBAT (Kang et al., 2015. PeerJ). The relative abundance of a given genome bin was calcul ated as its sequencing coverage divided by the total sequencing coverage of all genome bins in the corresp onding sample. Tengchong samples are described in Hua et al., 2018

File Name: Supplementary Data 3
Description: *Plain text file***.** Archaea tree file for the maximum likelihood phylogenetic tree of 3577 archae al and 40 bacterial genomes based on 37 concatenated protein-coding genes previously described in Baker et al., 2020 Nat. Microbiol (https://doi.org/10.1038/s41564-020-0715-z)

File Name: Supplementary Data 4
Description: *Excel File* Selected 250 publicly available TACK genomes including 15 Brockarchaeota gen omes reported in this study.

File Name: Supplementary Data 5
Description: *Excel File.* Sheet 1. Average amino acid identity (AAI) comparison of Brockarchaeota geno mes and phylogenetically related TACK phyla. Sheet 2. Selected TACK genomes closely related to Brock archaeota genomes. (Geoarchaeota, Aigarchaeota, Korarchaeota and Thaumarchaeota). See Supplementar y Figure 1. The color code indicates the AAI (%) from 0-100 (green to red respectively).

File Name: Supplementary Data 6
Description: *Excel File*, Metabolism sheet: Overview of key metabolic genes found in Brockarchaeota ge nomes using KofamKOALA. Only hits above the predefined threshold for individual KOs were sel ected. Raw sheet contains the raw annotations obtained with KofamKOALA including the specif ic scaffolds for each annotation. MT system sheet contains the

selected genes for the MT system and folD proteins detected with Interproscan v5.31-70.0. Metaboli c Marker genes sheet contains the refence and absence profile of selected marker genes obtained from M ETABOLIC (Zhou et al., 2019). MEBS completeness sheet shows the completeness of PFAM do mains in TACK genomes showed in Figure 1. MEBS mapping File sheet contains the full repertoire of prot ein domains involved in specific metabolic processes.

File Name: Supplementary Data 7
Description: *Plain text file***.** Fasta file containing the 16S rRNA gene sequences identified in Brockarchaeota MAGs. Sequences were extracted using and barrnap (https://github.com/tseemann/barrnap, v0.8, settings: --kingdom arc/bac --lencutoff 0.2 --reject 0.3 --evalue 1e-05).

File Name: Supplementary Data 8
Description: *Excel file* Metadata and geographic locations of 16S rRNA gene sequences related to Br ockarchaeota genomes obtained from the Integrated Microbial Genomes and Microbiomes (IMG/M) datab ase (https://img.jgi.doe.gov/).

File Name: Supplementary Data 9
Description: *Excel file Excel file*. Predicted transcription units of the scaffolds were methanol-cobalamin methyltransferases (MtaB), trimethylamine-corrinoid protein methyltransferase (MttB) and B 12-binding corrinoid proteins were found in Brockarchaeota genomes. The operon prediction based on int ergenic distance of neighboring genes as well as the functional relationships of their protein coding prod ucts was computed with Operon Mapper (Taboada et al., 2018).

File Name: Supplementary Data 10
Description: *Excel file***.** Description of the common core marker genes specific to methanogenic, anaerobic methanotrophic and short-chain alkane-oxidizing archaea described in Borrel et al., 2019, across the TACK superphylum. Sheet 2 includes the raw data with the scaffolds encodi ng the specific marker genes and corresponding E values. Presence and absence profile of common core m arker genes specific to methanogenesis across the TACK superphylum. Genes include those associated to methanogenesis, anaerobic methanotrophic and short-chain alkane-oxidizing archaea described in Borrel et al., 2019. For comparison purposes the following known methanogenic archaea were included: *Candidatus Methanoplasma termitum* that lacks the entire pathway for $CO_2$ reduction to methyl coenzyme M and prod uces methane by hydrogen-dependent reduction of methanol or methylamine (Methanomassiliicoccales), *M ethanosphaera stadtmanae* (Methanobacteriales) that can generate methane only by the reduction of meth anol with $H_2$ and is dependent on acetate as a carbon source, and *Methanosarcina acetivoran*s (Methanosar cinales). Predicted transcription units of the scaffolds were methylenetetrahydrofolate reductase (NADPH) [EC:1.5.1.20] was found in Brockarchaeota genomes (JZZ-4 and JZ-1.89 sheets respectively). The operon prediction based on intergenic distance of neighboring genes as well as the functional relationships of their protein coding products was computed with Operon Mapper(Taboada et al., 2018).

File Name: Supplementary Data 11
Description: *Excel file*. Metagenomic screening of Methyl-coenzyme M reductase (MCR) genes in metag enomic assemblies from which Brockarchaeota genomes where recovered.

File Name: Supplementary Data 12
Description: *Excel File*. Total number of carbohydrate-active enzymes (CAZymes) detected in Brockarchaeota and TACK superphylum. Sheet 1. Number of total CAZYmes in Brockarchaeota genomes. Sheet 2 contains the raw output from the dbCAN webserver and PSORT results. Sheet 3 Comparison of unique and shared CAZYmes across members of the TACK

superphylum. Only hits detected in more than two tools where selected for domain assignment. Total numb er of carbohydrate-binding modules (CBMs), carbohydrate esterases (CE), glycoside hydrolases GH) and p olysaccharide lyases (PL). Subcellular localization for CEs, GHs and PLs was determined using PSORTb.

File Name: Supplementary Data 13
Description: *Excel file* .Total number of peptidases detected in each Brockarchaeota genome. Total number of peptidases identified using the MEROPS v12.1 peptidase database. Subcellular localization for individua l peptidases was determined using PSORTb.
