# Supplementary material for: Brockarchaeota, a novel archaeal phylum with unique and versatile carbon cycling pathways
Source: Nat Commun. 2021 Apr 23;12:2404. doi: 10.1038/s41467-021-22736-6 (PMC8065059; doi:10.1038/s41467-021-22736-6)
Supplement: Supplementary file 16 — Reporting Summary [file 41467_2021_22736_MOESM16_ESM.pdf]

nature research

Corresponding author(s): Brett J. Baker
acidophile@gmail.com
Wen-Jun Li
liwenjun3@mail.sysu.edu.cn

Last updated by author(s): Feb 15, 2021

Reporting Summary

Nature Research wishes to improve the reproducibility of the work that we publish. This form provides structure for consistency and transparency in reporting. For further information on Nature Research policies, see our Editorial Policies and the Editorial Policy Checklist.

Statistics

For all statistical analyses, confirm that the following items are present in the figure legend, table legend, main text, or Methods section.

n/a	Confirmed	
☒	☐	The exact sample size (*n*) for each experimental group/condition, given as a discrete number and unit of measurement
☒	☐	A statement on whether measurements were taken from distinct samples or whether the same sample was measured repeatedly
☒	☐	The statistical test(s) used AND whether they are one- or two-sided<br>*Only common tests should be described solely by name; describe more complex techniques in the Methods section.*
☒	☐	A description of all covariates tested
☒	☐	A description of any assumptions or corrections, such as tests of normality and adjustment for multiple comparisons
☒	☐	A full description of the statistical parameters including central tendency (e.g. means) or other basic estimates (e.g. regression coefficient) AND variation (e.g. standard deviation) or associated estimates of uncertainty (e.g. confidence intervals)
☒	☐	For null hypothesis testing, the test statistic (e.g. *F*, *t*, *r*) with confidence intervals, effect sizes, degrees of freedom and *P* value noted<br>*Give P values as exact values whenever suitable.*
☒	☐	For Bayesian analysis, information on the choice of priors and Markov chain Monte Carlo settings
☒	☐	For hierarchical and complex designs, identification of the appropriate level for tests and full reporting of outcomes
☒	☐	Estimates of effect sizes (e.g. Cohen's *d*, Pearson's *r*), indicating how they were calculated

Our web collection on statistics for biologists contains articles on many of the points above.

Software and code

Policy information about availability of computer code

Data collection	The deep -sea MAGs (B48_G17 and B27_G9) are described in Dombrowski et al., 2018. Briefly Libraries for paired-end Illumina (HiSeq–2500 1TB) sequencing were prepared by the Joint Genome Institute (JGI). Sequencing was performed on an Illumina HiSeq 2500 machine using the paired-end 2x125 bp run-type mode. All runs combined provided a total of ~280 gigabases of sequencing data. Quality control and sequence assembly were performed by JGI. Briefly, sequences were trimmed and screened for low quality sequences using bbtools (https://jgi.doe.gov/data-and-tools/bbtools/) and assembled using megahit v1.0.6 using the following options: --k-list23,43,63,83,103,123. For further binning, only scaffolds ≥ 2000 bps were included. Metagenomic binning was performed on individual assemblies using the binning tools ESOM, Anvi'o (v2.2.2)67 and Metabat (v1)68. ESOM bins were extracted using getClassFasta.pl and the command -loyal 51. Anvi'o was run with default parameters and metabat was run using the following settings: --minProb 75 --minContig 2000 --minContigByCorr 2000. Results from the three different binning tools were combined using DAS Tool (version 1.0) as follows: DAS_Tool.sh -i Anvio_contig_list.tsv, Metabat_contig_list.tsv, ESOM_contig_list.tsv -l Anvio, Metabat, ESOM -c scaffolds.fasta --write_bins 1.<br>For the hot spring MAGs:<br>Eight MAGs (DRTY-1.18, DRTY-6.80, DRTY-6.200, DRTY7.37, JZ-1.89, JZ-2.136, JZZ_4 and DRTY7) were recovered from hot springs in Yunnan, China. Five additional MAGs (QC4_43, QC4_48, GD2_1_47_42, QZM_A2, QZM_A3) were reconstructed from hot springs in Tibet. Sequencing was done on an Illumina HiSeq4000 (Beijing Novogene Bioinformatics Technology Co., Ltd). These samples were assembled using metaSPADES (version 3.9.1), with a k-mer set of "21, 33, 55, 77, 99, 127". For each sample only scaffolds larger than 2500 bp were binned using MetaBAT (v.2.12.1) with default parameters, considering both tetranucleotide frequencies (TNF) and scaffold coverage information. The scaffolds from the obtained bins and the unbinned scaffolds were visualized using ESOM with a minimum length of 2500 bp and maximum length of 5000 bp as previously described69 and the bins were modified by removing any out-of-range scaffolds (indicated by sequence points) or adding any unbinned scaffolds using ESOM related scripts43. MAGs from Tibet hot springs with scaffolds ≥ 1000 bp were uploaded to ggKbase (http://ggkbase.berkeley.edu/), and the bins from ESOM analyses were evaluated and modified manually at ggKbase based on GC content, coverage and taxonomic information of scaffolds. MAGs from Tengchong hot springs were reassembled using SPAdes (version3.9.1) under the "careful" mode with the same k-mers. During this step, the reads used for the assemblies were recruited by mapping clean reads to the curated

genome bins using BBmap (v35.85; http://sourceforge.net/projects/bbmap/). The accuracy of all the MAGs was evaluated by calculating the percentage of completeness and gene duplications using CheckM lineage_wf (v1.0.5)

Data analysis

Data was analyzed using the following published softwares:
- MetaBAT v2.12.1
- Bowtie2 v2.3.5.1
- dbcan2 (http://bcb.unl.edu/dbCAN2/)
- Interproscan v5.31.70
- HMMER 3.1b2
- MEBSv1
- Psort v3.0
- IQ-TREE v. 1.6.1
- DIAMOND v0.9.24.125
- RAxML v8.2.10
- Geneious Prime 2020.0.5
- KofamKOALA KEGG release 96.0 (https://www.genome.jp/tools/kofamkoala/)
- ARB v. 2.5b
- Operon mapper (https://biocomputo.ibt.unam.mx/operon_mapper/)
- SeqKit v0.14.0
- CheckM v1.0.5 (https://ecogenomics.github.io/CheckM/)
- CompareM v0.0.23 (https://github.com/dparks1134/CompareM)
- Prodigal v2.6.3 (https://github.com/hyattpd/Prodigal)
- Barrnap v0.9 (https://github.com/tseemann/barrnap)
- Bowtie2
- MAFFT v7.450
-MUSCLE v3.8.425

Customs scripts and command lines

- Useful scripts repository (https://github.com/valdeanda/Useful_scripts)
- IMGap (https://github.com/valdeanda/IMG_annotation)

Data Visualization:
- Adobe Illustrator CC 2020 (24.0)
- BioRender.com 2021
- Itol v5 (https://itol.embl.de/)

For manuscripts utilizing custom algorithms or software that are central to the research but not yet described in published literature, software must be made available to editors and reviewers. We strongly encourage code deposition in a community repository (e.g. GitHub). See the Nature Research guidelines for submitting code & software for further information.

Data

Policy information about availability of data

All manuscripts must include a data availability statement. This statement should provide the following information, where applicable:
- Accession codes, unique identifiers, or web links for publicly available datasets
- A list of figures that have associated raw data
- A description of any restrictions on data availability

Data availability. The final assembled and annotated genomic sequences of Brockarchaeota from deep sea sediments (B27_G9 and B48_G17) have been deposited in NCBI under BioProject ID PRJNA362212: BioSample id SAMN09215183 and SAMN09214986 respectively. Sequence data and sample information of Brockarchaeota from hot springs are available at NCBI under Bio Project ID PRJNA544494. All the NCBI accession numbers for the MAGs described in this study are provided in Supplementary Data 1. The datasets generated during and/or analyzed during the current study that has not been provided in supplementary data are available from the corresponding author on request.

Databases used to analyze data and or against which data was compared:
- nr (ftp://ftp.ncbi.nlm.nih.gov/)
- UniProtKB (https://www.uniprot.org/)
-Pfam (https://pfam.xfam.org/)
- HydDB (https://services.birc.au.dk/hyddb/)
- Carbohydrate-Active enZYmes (CAZYmes) database (http://www.cazy.org/)
- MEROPS peptidase database (https://www.ebi.ac.uk/merops/)
- Metacyc Metabolic Pathway Database (https://metacyc.org/)
- KEGG (https://www.genome.jp/kegg/)
- COGs and arCOGs (ftp://ftp.ncbi.nih.gov/pub/wolf/COGs/arCOG)
- IMG/MER (https://img.jgi.doe.gov/mer/)

Field-specific reporting

Please select the one below that is the best fit for your research. If you are not sure, read the appropriate sections before making your selection.

☐ Life sciences ☐ Behavioural & social sciences ☒ Ecological, evolutionary & environmental sciences

For a reference copy of the document with all sections, see nature.com/documents/nr-reporting-summary-flat.pdf

Ecological, evolutionary & environmental sciences study design

All studies must disclose on these points even when the disclosure is negative.

Study description	Deep sea-derived MAGs (B48_G17 and B27_G9) analyzed here are part of a larger study described in Dombrowski et al., 2018 that aims to characterize the geochemical conditions and microbial community of<br>Guaymas Basin (GB) hydrothermal vent sediments (Gulf of California, Mexico). DNA was extracted from sediment samples using the MO BIO – PowerMax Soil DNA Isolation kit and sent to the Joint Genome Institute (JGI) for sequencing. A lane of Illumina reads (HiSeq–2500 1TB, read length of 2x151 bp) were generated for samples. De novo Assembly and Binning was used to create metagenome-assembled genomes (MAGs). Phylogeney was preformed to identify genomes of interests and two novel genomes were chosen for further metabolic analysis in this study
Research sample	Two MAGs (B48_G17 and B27_G9) were obtained from Guaymas Basin sediments (Gulf of California; 27°N0.388, 111°W24.560) and were obtained as part of a larger study of these hydrothermal marine sediments25. Both samples were collected from the same location but G9 was sampled from 0-3 cm and G17 form 12-15 cm depth. Eight MAGs (DRTY-1.18, DRTY-6.80, DRTY-6.200, DRTY7.37, JZ-1.89, JZ-2.136, JZZ_4 and DRTY7) were recovered from hot springs in Yunnan, China collected in January of 2016 and May of 2017 in several hot springs (Supporting Data 1). Five additional MAGs (QC4_43, QC4_48, GD2_1_47_42, QZM_A2, QZM_A3) were reconstructed from hot springs in Tibet in August of 2016. Sequencing was done on an Illumina HiSeq4000 (Beijing Novogene Bioinformatics Technology Co., Ltd).<br>Five hot spring sediment samples collected from Tibet Plateau (China) in August 2016 with Brockarchaeota spp. were included in this study for analyses. These samples have a temperature range of 61.8-69.5 °C. No replicates were performed.<br>Six hot spring sediment samples from five different places (JZ-1, JZ-2, DRTY-1, DRTY-6, and DRTY-7) located at the collision boundary between the India and Eurasia plates near Tengchong city in Yunnan province (China) were collected in Jan 2016 and May 2017 respectively. The temperatures for these samples are ranging from 55.8-86.5ºC. No replicates were performed.
Sampling strategy	For the Guaymas samples, the samples were taken based on distance to previously studied hydrothermal vents and the presence/abscence of a microbial mat.<br>The sediment samples from Tibet Platea, were collected from the hot spring pools using a sterile iron spoon into 50 ml sterile tubes, transported to the lab on dry ice, and stored at -80 °C for DNA extraction.<br>The Tengchong samples were collected using sterile spatulas and spoons and stored in liquid nitrogen before transporting to the lab.
Data collection	Guaymas Basin sediment samples were collected from the Gulf of California (27°N0.388, 111°W24.560) at a depth of approximately 2,000 m below the water surface. The sediment cores from which the two deep sea MAGs were binned from were collected during Alvin dive 4571_4 in 2009 using polycarbonate cores (45-60 cm in length, 6.25 cm interior diameter), subsampled into cm layers under N2 gas in the ship's laboratory and immediately frozen at -80°C. Details on the sampling site and metagenomic sequencing effort is provided in Dombrowski et al., 2018.Sample site photos were compiled from the Alvin frame grabber site (http://4dgeo.whoi.edu/alvin)<br>Temperature, dissolved oxygen (DO) and pH were determined in situ and the other physicochemical parameters were analyzed in the laboratory by H.C.J for the sediment samples from Tibet Platea,<br>For the Tengchong samples Temperature, dissolved oxygen (DO) and pH were determined in situ and the other physicochemical parameters were analyzed in the laboratory. Community genomic DNA was extracted from approximately 20 g of sediment material using PowerSoil DNA Isolation kit (MoBio). DNA concentrations of the extract and constructed libraries (with insert size of 350 bp) were measured with a Qubit fluorometer. Metagenomic sequence data for the two samples are generated using Illumina Hiseq 4000 instruments at Beijing Novogene Bioinformatics Technology Co., Ltd (Beijing, China). The amount of raw sequence data was ~30 Gbp (2×150bp) for each sample
Timing and spatial scale	The deep sea guaymas data analyzed in this study was collected in December 2009 on Alvin dives 4571_4. These samples were chosen based on the presence of the novel phylum in the MAGs generated by De novo assembly and binning
Data exclusions	Data gathered from Guaymas samples are described in Dombrowski, N., Teske, A. P. & Baker, B. J. Extensive metabolic versatility and redundancy in microbially diverse, dynamic Guaymas Basin hydrothermal sediments. Nat. Commun (2018)<br>Data gathered from the Tibet and Tengchong samples are described in Chen et al. 2019 (https://doi.org/10.3389/fmicb.2019.00928) and and Hua et al. 2018 and 2019 (https://doi.org/10.3389/fmicb.2019.00928) respectively
Reproducibility	We have in detail described our methodological approaches for genome annotations as well as phylogenetic analyses to ensure reproducibility. We calculated branch support values for the different clusters obtained in our phylogenies that provide an indication as to how stable a given monophyletic group is. Considering this, our phylogenetic analyses are reproducible (when the same phylogenetic strategy is applied such as the same model of evolution etc). Genome annotations are based on inferences from various different databases. While this provides some level of confidence, annotations may have to be updated when new

	information is generated and deposited in databases.
Randomization	Since central tendencies and deviations between different treatments and controls have not been studied here, randomization and blinding are not applicable
Blinding	Blinding was not performed because it was not applicable to this study. This study was a survey of various populations, and was not dependent on the presence / absence of certain characteristics.

Did the study involve field work? ☒ Yes ☐ No

Field work, collection and transport

Field conditions	Hot spring MAGs Samples are from thermal habitats with temperature ranging from 60~98 °C and pH ranging from 6.0 to 9.6. Deep sea MAGs were obtained from Guaymas Basin sediments (Gulf of California; 27°N0.388, 111°W24.560) and were obtained as part of a larger study of these hydrothermal marine sediments.
Location	Deep sea MAG (B48_G17 and B27_G9s are from the Guaymas Basin (Gulf of California; 27°N0.388, 111°W24.560), Mexico. were recovered from hot springs in Yunnan, China collected in January of 2016 and May of 2017 in several hot springs (Supporting Data 1). Five additional MAGs (QC4_43, QC4_48, GD2_1_47_42, QZM_A2, QZM_A3) were reconstructed from hot springs in Tibet
Access & import/export	N/A
Disturbance	No disturbance

Reporting for specific materials, systems and methods

We require information from authors about some types of materials, experimental systems and methods used in many studies. Here, indicate whether each material, system or method listed is relevant to your study. If you are not sure if a list item applies to your research, read the appropriate section before selecting a response.

Materials & experimental systems

n/a	Involved in the study	
☒	☐	Antibodies
☒	☐	Eukaryotic cell lines
☒	☐	Palaeontology and archaeology
☒	☐	Animals and other organisms
☒	☐	Human research participants
☒	☐	Clinical data
☒	☐	Dual use research of concern

Methods

n/a	Involved in the study	
☒	☐	ChIP-seq
☒	☐	Flow cytometry
☒	☐	MRI-based neuroimaging
